# Cardiac Fibrosis and Innervation State in Uncorrected and Corrected Transposition of the Great Arteries: A Postmortem Histological Analysis and Systematic Review

**DOI:** 10.3390/jcdd10040180

**Published:** 2023-04-20

**Authors:** Leo J. Engele, Roel L. F. van der Palen, Anastasia D. Egorova, Margot M. Bartelings, Lambertus J. Wisse, Claire A. Glashan, Philippine Kiès, Hubert W. Vliegen, Mark G. Hazekamp, Barbara J. M. Mulder, Marco C. De Ruiter, Berto J. Bouma, Monique R. M. Jongbloed

**Affiliations:** 1 Center for Congenital Heart Disease Amsterdam-Leiden (CAHAL), Department of Clinical and Experimental Cardiology, Amsterdam Cardiovascular Sciences, Heart Centre, Amsterdam UMC, University of Amsterdam, 1105 AZ Amsterdam, The Netherlands; 2 Netherlands Heart Institute, 3511 EP Utrecht, The Netherlands; 3 Center for Congenital Heart Disease Amsterdam-Leiden (CAHAL), Department of Pediatric Cardiology, Leiden University Medical Center, 2333 ZA Leiden, The Netherlands; 4 Center for Congenital Heart Disease Amsterdam-Leiden (CAHAL), Department of Cardiology, Leiden University Medical Center, 2333 ZA Leiden, The Netherlands; 5 Center for Congenital Heart Disease Amsterdam-Leiden (CAHAL), Department of Anatomy and Embryology, Leiden University Medical Center, 2333 ZA Leiden, The Netherlands; 6 Center for Congenital Heart Disease Amsterdam-Leiden (CAHAL), Department of Cardiothoracic Surgery, Leiden University Medical Center, 2333 ZA Leiden, The Netherlands

**Keywords:** transposition of the great arteries, Mustard Senning procedure, arterial switch operation, myocardial fibrosis, innervation

## Abstract

In the transposition of the great arteries (TGA), alterations in hemodynamics and oxygen saturation could result in fibrotic remodeling, but histological studies are scarce. We aimed to investigate fibrosis and innervation state in the full spectrum of TGA and correlate findings to clinical literature. Twenty-two human postmortem TGA hearts, including TGA without surgical correction (n = 8), after Mustard/Senning (n = 6), and arterial switch operation (ASO, n = 8), were studied. In newborn uncorrected TGA specimens (1 day–1.5 months), significantly more interstitial fibrosis (8.6% ± 3.0) was observed compared to control hearts (5.4% ± 0.8, *p* = 0.016). After the Mustard/Senning procedure, the amount of interstitial fibrosis was significantly higher (19.8% ± 5.1, *p* = 0.002), remarkably more in the subpulmonary left ventricle (LV) than in the systemic right ventricle (RV). In TGA-ASO, an increased amount of fibrosis was found in one adult specimen. The amount of innervation was diminished from 3 days after ASO (0.034% ± 0.017) compared to uncorrected TGA (0.082% ± 0.026, *p* = 0.036). In conclusion, in these selected postmortem TGA specimens, diffuse interstitial fibrosis was already present in newborn hearts, suggesting that altered oxygen saturations may already impact myocardial structure in the fetal phase. TGA-Mustard/Senning specimens showed diffuse myocardial fibrosis in the systemic RV and, remarkably, in the LV. Post-ASO, decreased uptake of nerve staining was observed, implicating (partial) myocardial denervation after ASO.

## 1. Introduction

**Figure 1:**
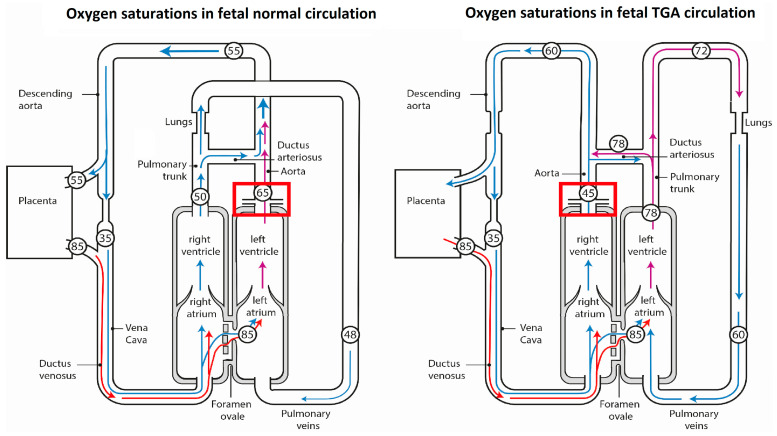
Normal circulation (**left panel**) and circulation in a fetus with TGA (**right panel**). The numbers indicate percentage of oxygen saturations in the cardiac chambers and great vessels, and are based on estimated values of umbilical blood flow and left and right ventricular output in the fetus [2]. In TGA, the subpulmonary left ventricle ejects blood into the pulmonary circulation so that blood perfusing the lungs and passing through the ductus arteriosus has a relatively high oxygen saturation. Blood entering the ascending aorta and coronary arteries has an oxygen saturation considerably lower than in the normal circulation (45% versus 65%, indicated by red squares). High oxygen saturations are illustrated by red arrows and lower oxygen saturations by purple and blue arrows. Abbreviations: TGA, transposition of the great arteries. The figure is modified and adapted from Rudolph et al. [2]. Copyright 2023 by Copyright Clearance Center.

Transposition of the great arteries (TGA) is a complex congenital heart malformation and affects approximately 3 in 10,000 live births [1]. The morphological key feature of TGA is ventriculoarterial discordance, with the aorta arising from the morphological right ventricle (RV) and the pulmonary artery from the morphological left ventricle (LV). TGA is compatible with fetal survival and normal body growth; however, the ventriculoarterial discordance and accompanying lesions are associated with altered hemodynamics and oxygen saturation in the fetal circulation [2]. Although venous flow patterns are similar to normal in fetal TGA patients, higher oxygen saturations (>75%) in TGA are presumed to be present in the pulmonary artery and ductus arteriosus as compared to the normal fetal heart (50%) and lower oxygen saturations in the ascending aorta and coronary arteries (45% versus 65%) [2] (Figure 1). As a consequence, to provide the same oxygen delivery as in the normal fetus, coronary perfusion would have to be substantially higher. We hypothesize that this lower coronary oxygen saturation in utero might induce fibrotic remodeling of the myocardium in the TGA circulation that may persist after birth. 

Recognition of myocardial remodeling is of great importance as previous studies in non-congenital heart disease reported that diffuse myocardial fibrosis is associated with worsening ventricular function and increased ventricular stiffness [3,4]. Previous cardiac magnetic resonance studies (CMR) in TGA patients after Mustard/Senning correction or arterial switch operation (ASO) have reported prolonged T1 relaxation times and increased extracellular volume (ECV) volume [5,6], thereby suggesting the presence of myocardial remodeling even after surgical correction. However, histological studies on myocardial fibrosis in TGA to substantiate these findings are scarce, and therefore, less is known about the time span during which remodeling occurs and whether myocardial remodeling results in a significant amount of myocardial fibrosis.

Furthermore, TGA patients are nowadays corrected using the ASO and may be denervated due to the transection of the nerves along the great arteries and reimplantation of the coronary arteries. As innervation might influence myocardial maturation [7], partial (transient) denervation after the ASO could potentially affect ventricular function. This information might also be of clinical relevance as patients with myocardial ischemia could be asymptomatic due to denervation. 

The aim of the current study was to perform a histological assessment of myocardial fibrosis, myocardial structure, and myocardial innervation in uncorrected TGA postmortem, post-Mustard/Senning and post-ASO hearts and to correlate these findings to current literature.

## 2. Materials and Methods

### 2.1. Study Design

This postmortem study was performed in accordance with the local biobank and ethics committee of the Leiden University Medical Center and with Dutch regulations for proper use of human tissue for medical research purposes. Postmortem heart specimens from the Leiden Collection of Congenital Malformations, that is part of the Biobank Congenital Heart Disease of the Leiden University Medical Center, were included in this study. The Leiden Collection of Congenital Malformations is part of the institutional Biobank Congenital Heart Disease, that is approved by the Medical Ethical Committee Leiden-Den Haag-Delft. The local scientific committee and Medical Ethical Committee Leiden-Den Haag-Delft approved the working protocol of the current study (protocol number RP23.006).

### 2.2. Selection Criteria Postmortem Hearts

**Figure 2:**
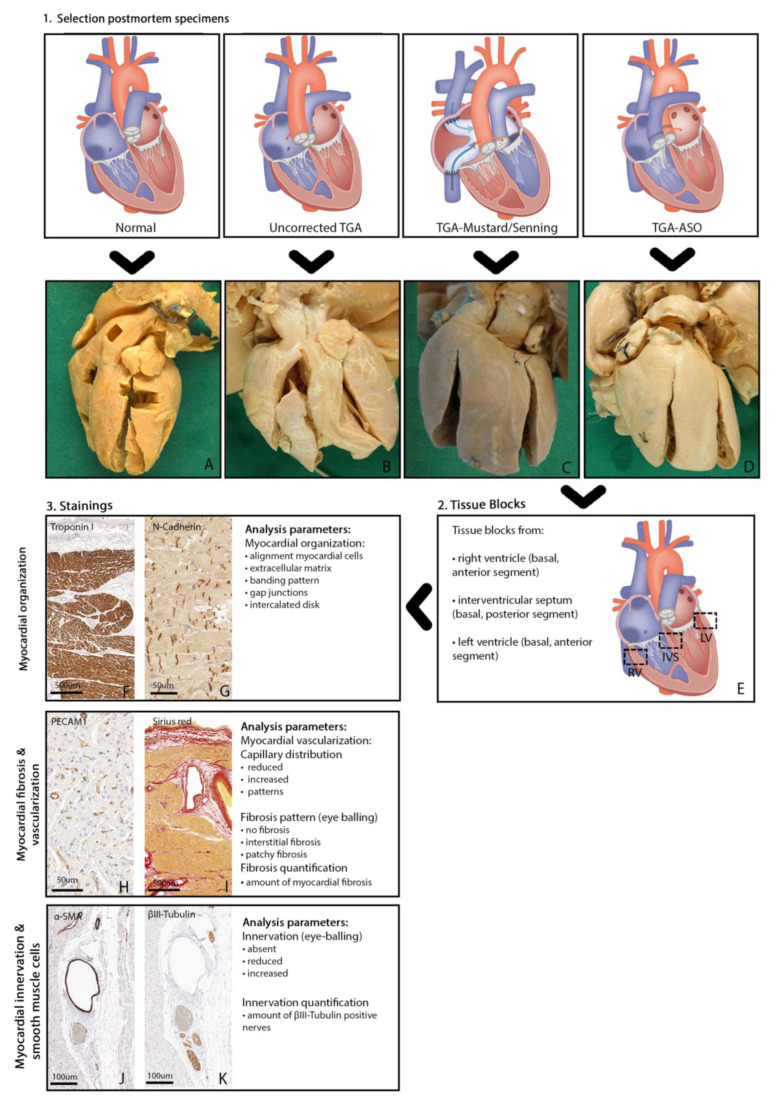
Illustration study design. Study design: illustration of all consecutive steps. 1. Selection postmortem specimens: postmortem specimens were obtained from the Leiden Collection of Congenital Malformations, including the following groups: normal specimens (**A**), uncorrected TGA specimens (**B**), TGA-Mustard/Senning specimens (**C**) and TGA-ASO specimens (**D**). 2. Tissue blocks: transmural tissue blocks were obtained from three different sites in each specimen (**E**); right ventricle, interventricular septum, and left ventricle. 3. Stainings: the following (DAB) stainings were performed: cardiac troponin-I (**F**) (myocardial organization), N-Cadherin (**G**) (myocardial organization), PECAM1 (**H**) (myocardial vascularization), Picrosirius Red (**I**) (myocardial fibrosis), α-SMA (**J**) (smooth muscle cells) and βIII-tubulin (**K**) (myocardial innervation). Abbreviations: ASO, arterial switch operation; DAB, 3-30diaminobenzidine tetrahydrochloride; IVS, interventricular septum; LV, left ventricle; PECAM1, platelet endothelial cell adhesion molecule-1; RV, right ventricle; α-SMA, alpha smooth muscle actin; TGA, transposition of the great arteries.

Specimens were selected based on age, sex, and, in case of operation, duration of survival after operation (naturally delimited by the availability of specimens). As we aimed to investigate the presence of myocardial fibrosis in newborn TGA patients and during aging, we included pediatric TGA postmortem hearts without surgical intervention (uncorrected TGA) of various ages. We also included one available uncorrected adult TGA postmortem heart (20 years). Secondly, we included postmortem TGA hearts who survived at least six months after atrial switch operation (TGA-Mustard/Senning) to investigate the impact of physiological correction on the presence of fibrosis in the subpulmonary LV and systemic RV. Finally, we included all available TGA specimens after arterial switch operation (TGA-ASO) to investigate the presence of fibrosis and whether histological denervation occurs after transection of the great arteries and reimplantation of the coronary arteries. Histological findings from TGA specimens were compared with postmortem hearts without congenital heart defects (control group). Hearts from the control group (i.e., without congenital heart disease) that on further inspection showed signs of heart failure or ventricular overload, were excluded. Figure 2 demonstrates the study design with the different groups for histological assessment (Figure 2A–D), as well as the histological readout parameters.

### 2.3. Tissue Blocks of Postmortem Hearts

From each postmortem heart, transmural tissue blocks were obtained at the following sites: RV basal anterior segment, LV basal anterior segment, and septal basal posterior segment (See Figure 2E). Depending on the size of the postmortem hearts, tissue blocks varied between 5 mm and 15 mm. 

### 2.4. Immunohistochemical Staining and Analysis

Biopsies were embedded in paraffin, and 5 μm sections were mounted on KP-Silane adhesive glass slides (Klinipath). Sections were stained with primary antibodies, followed by a second incubation with an antibody coupled to peroxidase. For detection, we used 3-30diaminobenzidine tetrahydrochloride (DAB). To examine myocardial structure and organization, sections were stained with cardiac troponin-I (Figure 2F), and we used N-Cadherin (NCAD)(Figure 2G) to assess the presence of gap junctions and adherence junctions. Vascular endothelial cells were assessed with platelet endothelial cell adhesion molecule-1 (PECAM-1) staining (Figure 2H). Picrosirius Red (cross-stained with Weigert’s Hematoxylin) staining was used to visualize myocardial collagen (Figure 2I). For the assessment of smooth muscle cells, we used α smooth muscle actin (α-SMA) staining (Figure 2J). The presence of subepicardial and myocardial autonomic nerves was examined using βIII-tubulin, a general nerve marker (Figure 2K). Supplemental Table S1 shows all antibodies used for immunohistochemical analysis. For each glass, high-resolution photomicrographs were taken with a 3DHistech Pannoramic 250 Flash III digital scanner at 20× magnification. 

### 2.5. Myocardial Fibrosis Patterns and Quantification

**Figure 3:**
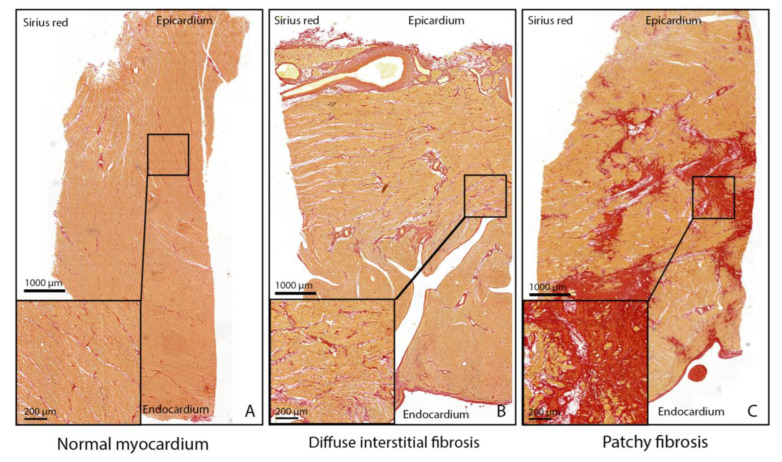
Myocardial fibrosis patterns in control and transposition of the great arteries. Myocardial fibrosis patterns using Sirius red staining. (**A**) Myocardium with normal pattern and amount of collagen. (**B**) Diffuse distribution of interstitial myocardial fibrosis. (**C**) Multiple areas with patchy fibrosis.

Myocardium was defined as normal if myocardial collagen was seen at the epicardial surface, at the endocardial surface, and around blood vessels with the distribution of linear collagen parallel to cardiomyocytes, not entirely surrounding the cardiomyocytes [8] (Figure 3A). Transmural interstitial fibrosis was defined as an increased amount of linear collagen parallel or surrounding the cardiomyocytes [8] (Figure 3B). The architecture of fibrosis was defined as diffuse interstitial fibrosis (Figure 3B) or patchy fibrosis [9] (Figure 3C).

In all biopsies stained with Picrosirius Red, the amount of fibrosis was quantified using custom software (Python 2.7), as previously described [9]. Each pixel was classified as red (collagen), yellow (myocardium), black (nuclei), or white (non-staining tissue). As our objective was to assess the amount of transmural myocardial collagen, the endocardium, and epicardium were excluded in the quantification to avoid bias from postmortem hearts with an increased thickness of the endocardium or epicardium. The amount of collagen was calculated as a percentage of the total tissue area by dividing the number of red pixels by the sum of red, black, and yellow pixels. With this method, we corrected for differences between tissue block sizes (Supplemental Figure S1). Quantification of Picrosirius Red staining in postmortem hearts without congenital heart disease was used as a reference value for healthy myocardium. 

### 2.6. Myocardial Innervation: βIII-Tubulin Patterns and Quantification

In tissue blocks stained to detect βIII-tubulin, photomicrographs were taken in areas around cross-sectioned coronary vessels, visually assessed, and compared between TGA-ASO and carefully matched sections in uncorrected TGA postmortem hearts. The amount of βIII-tubulin-positive nerves was quantified using custom software (Python 2.7). A threshold for brown color was set, and the percentage of brown pixels was calculated by dividing the number of brown pixels by the total amount of pixels within the tissue block.

### 2.7. Literature Review

To correlate the findings of our study with current literature, a comprehensive literature review with the subjects 1. myocardial fibrosis in TGA and 2. myocardial innervation in TGA post-ASO was performed in PubMed, Embase, Web of Science, Cochrane Library, and Emcare. Two separate search strategies were executed with the following keywords: Search strategy 1: Transposition of the great arteries, Mustard/Senning operation, arterial switch operation, myocardial fibrosis. Search strategy 2: arterial switch operation and innervation (See Text S1 and Text S2 in the online supplement for the complete queries). Articles with the inclusion of TGA patients, with or without surgical correction, and investigation on the presence of myocardial remodeling in TGA or cardiac innervation post-arterial switch operation were included, and results were systematically reviewed. 

### 2.8. Statistical Analysis

Data analysis was performed in IBM SPSS Statistics version 25 (SPSS Inc., Chicago, IL, USA). Normally distributed variables were reported as mean ± standard deviation and not normally distributed variables as the median and interquartile range (IQR1–IQR3). Where appropriate, differences between continuous data were compared by Student’s t-test or Mann–Whitney U test. A *p*-value of <0.05 was considered statistically significant. 

## 3. Results

### 3.1. Postmortem Hearts

Four subgroups of postmortem hearts were included in the Leiden Collection of Congenital Malformations. Subgroup 1: TGA hearts (n = 8) without surgical correction (uncorrected TGA) consisting of seven pediatric hearts (age range: 1 day–5.0 years) and one adult postmortem heart (20 years). Subgroup 2: TGA-Mustard/Senning postmortem hearts (n = 6), including five pediatric hearts (age range: 1–5.0 years) and one adult TGA-Mustard/Senning postmortem heart (age 33 years). Time until surgical correction (Mustard or Senning procedure) was 12 ± 6.8 months, and the median time between surgical correction and death was 3 years (range 6 months–33 years). Subgroup 3: TGA hearts after ASO (n = 8). Of these, seven pediatric postmortem hearts (age range 1 day–4.5 years) survived only a short time after ASO (time range: 1 day–15 days), and one adult postmortem heart survived 19 years after ASO. Subgroup 4: Pediatric control hearts (n = 3) without congenital heart disease (age range: 1 day–1.5 years). In these 25 postmortem hearts, a total of 75 tissue blocks were taken for further analysis. Characteristics of the TGA postmortem hearts are summarized in Table 1.

### 3.2. Myocardial Fibrosis

#### 3.2.1. Myocardial Fibrosis in Control Group

**Figure 4:**
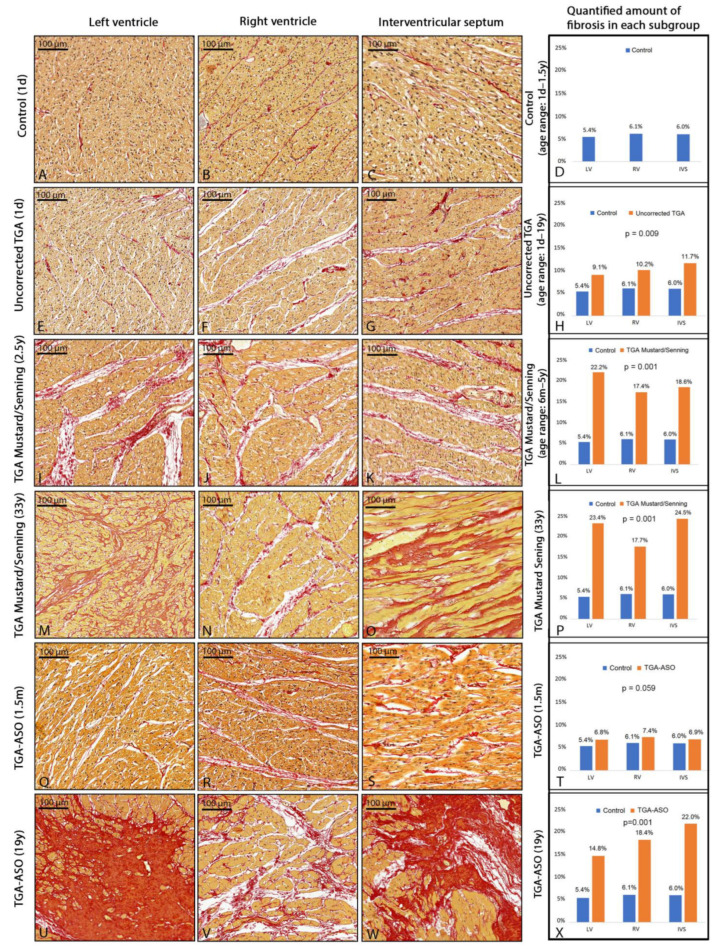
Patterns and amount of fibrosis in control, uncorrected TGA, TGA-Mustard/Senning, and TGA-ASO specimens. Fibrosis patterns and quantification in postmortem hearts (Sirius red staining). Normal myocardium at 1 day (**A**–**C**) and mean reference values in 3 control hearts without congenital heart disease (**D**). Normal myocardium of the subpulmonary LV (**E**) and increased amount of interstitial fibrosis in systemic RV (**F**) and interventricular septum (**G**) in uncorrected TGA at 1 day. Mean amount of fibrosis in 8 uncorrected TGA specimens (**H**). Diffuse interstitial fibrosis in 2.5-year-old TGA-Mustard/Senning specimen (**I**–**K**). Mean amount of fibrosis in 5 pediatric TGA-Mustard/Senning specimens (**L**). Patchy fibrosis (**M**) in the subpulmonary LV and interstitial fibrosis in the systemic RV (**N**) and interventricular septum (**O**) in TGA-Mustard/Senning specimen at the age of 33 years. The quantified amount of fibrosis in TGA-Mustard/Senning specimen at the age of 33 years (**P**). Normal myocardium of the systemic LV (**Q**) and interstitial fibrosis patterns at the subpulmonary RV (**R**) and interventricular septum (**S**) in TGA-ASO specimen at the age of 1.5 months. The quantified amount of fibrosis in TGA-ASO at the age of 1.5 months (**T**). Patchy fibrosis in the systemic LV (**U**) and interventricular septum (**W**) and interstitial fibrosis in subpulmonary RV (**V**). The quantified amount of fibrosis in TGA-ASO at the age of 19 years (**X**). Abbreviations: ASO, arterial switch operation; d, days; m, months; TGA, transposition of the great arteries; y, years.

All three postmortem hearts in the control group were devoid of interstitial or patchy fibrosis at the visual inspection (Figure 4A–C). The quantified mean amount of collagen in the control group (n = 3) was 5.4% ± 1.7 in the LV, 6.1% ± 2.0 in the RV, and 6.0% ± 1.8 in the interventricular septum (IVS). (Figure 4D). 

#### 3.2.2. Myocardial Fibrosis in Uncorrected TGA

Assessment of myocardial fibrosis in uncorrected TGA postmortem hearts showed an increased amount of fibrosis as compared to control hearts. Diffuse interstitial fibrosis was the most common pattern in 7/8 (88%) of the uncorrected TGA specimens. Of interest, the localization of diffuse interstitial fibrosis in 5 (63%) specimens was more pronounced in subendocardial/midventricular areas. The uncorrected TGA specimen at the age of 1 day old showed normal myocardium in the subpulmonary LV (Figure 4E) but increased fibrosis in the systemic RV (Figure 4F) and interventricular septum (Figure 4G). One postmortem heart (age 3 years) showed patchy subendothelial fibrosis in both the LV and RV. Categorized upon specimen age, the following mean cardiac fibrosis percentages were found: 6.7% ± 1.8 at 1 day, 8.0% ± 1.6 at 1 month, 7.5% ± 2.0 at 2.5 years, 7.7% ± 1.7 at 5.0 years, and 11.9% ± 3.5 at 18 years (Table 2). The amount of fibrosis was significantly higher in the uncorrected newborn (1 day–1.5 month) TGA specimens (8.6% ± 3.0) compared to control hearts of the same age (5.4% ± 0.8, *p* = 0.016). In uncorrected TGA, myocardial fibrosis was 9.1% ± 5.2 in the subpulmonary LV, 10.2% ± 3.7 in the systemic RV, and 11.7% ± 6.7 in the interventricular septum for all specimens combined (n = 8) and was significantly higher compared to controls (*p* = 0.009) (Figure 4H). The mean amount of fibrosis was 11.1% ± 6.8 in TGA specimens with the intact ventricular septum (IVS) (n = 3), 9.8% ± 2.2% with ventricular septum defect (VSD) (n = 3), and 10.0% ± 2.8 in double outlet right ventricle type taussig bing (TB-DORV, n = 2).

#### 3.2.3. Myocardial Fibrosis in TGA-Mustard/Senning

All TGA-Mustard/Senning postmortem specimens showed interstitial fibrosis patterns (Figure 4I–K). The quantified amount of fibrosis for all TGA-Mustard/Senning specimens was significantly higher compared to the control group (19.8% ± 5.1 versus 5.8% ± 1.0, respectively, *p* = 0.002). Categorized upon age, the amount of fibrosis at the age of 1 year, 1.8 years, 2.5 years, 3.5 years, 5 years, and 33 years was 27.3% ± 8.2, 12.9% ± 3.4, 15.7% ± 2.6, 21.5% ± 4.8, 19.6% ± 2.7, and 21.9% ± 4.4, respectively (Table 2). Comparison between the amount of fibrosis in the LV, RV, and IVS within the TGA-Mustard/Senning group showed that the amount of fibrosis in the pediatric specimens (n = 5) was 22.2% ± 8.8 at the subpulmonary LV, 17.4% ± 2.3 in the systemic RV and 18.6% ± 5.1 in the IVS, and was significantly increased compared to controls (*p* = 0.001) (Figure 4L). Interstitial fibrosis in both subpulmonary LV and systemic RV was, in most cases, located at the subendocardium or midventricular area. The adult TGA-Mustard/Senning specimen showed patchy fibrosis at the subpulmonary LV (Figure 4M) and interstitial fibrosis at the systemic RV (Figure 4N) and IVS (Figure 4O). TGA subtype analysis demonstrated that the mean amount of fibrosis was 16.1% in TGA-IVS (n = 3), 27.3% in TGA-VSD (n = 1), and 21.7% in TB-DORV (n = 2). 

#### 3.2.4. Myocardial Fibrosis in TGA-ASO Specimens

As most TGA-ASO postmortem specimens had died shortly after the ASO procedure, we only analyzed postmortem hearts that had survived at least 15 days after ASO to investigate the presence of myocardial fibrosis in TGA-ASO. One TGA-ASO postmortem heart (subtype: TGA-IVS) was operated at the age of 1 month and died 15 days after ASO. Sirius red staining showed diffuse interstitial fibrosis in the subpulmonary RV (Figure 4R) and IVS (Figure 4S). The amount of fibrosis in the systemic LV, subpulmonary RV, and interventricular septum was, respectively, 6.8%, 7.4%, and 6.9%, *p* = 0.059 (Figure 4T). The second ASO postmortem heart (TGA-IVS) survived 19 years after ASO, and patchy fibrosis was found in the LV (Figure 4U) and interventricular septum (Figure 4W), whereas the RV (Figure 4V) showed severe diffuse interstitial fibrosis. Unfortunately, no further clinical details were available. 

### 3.3. Myocardial Organization and Vascularization

**Figure 5:**
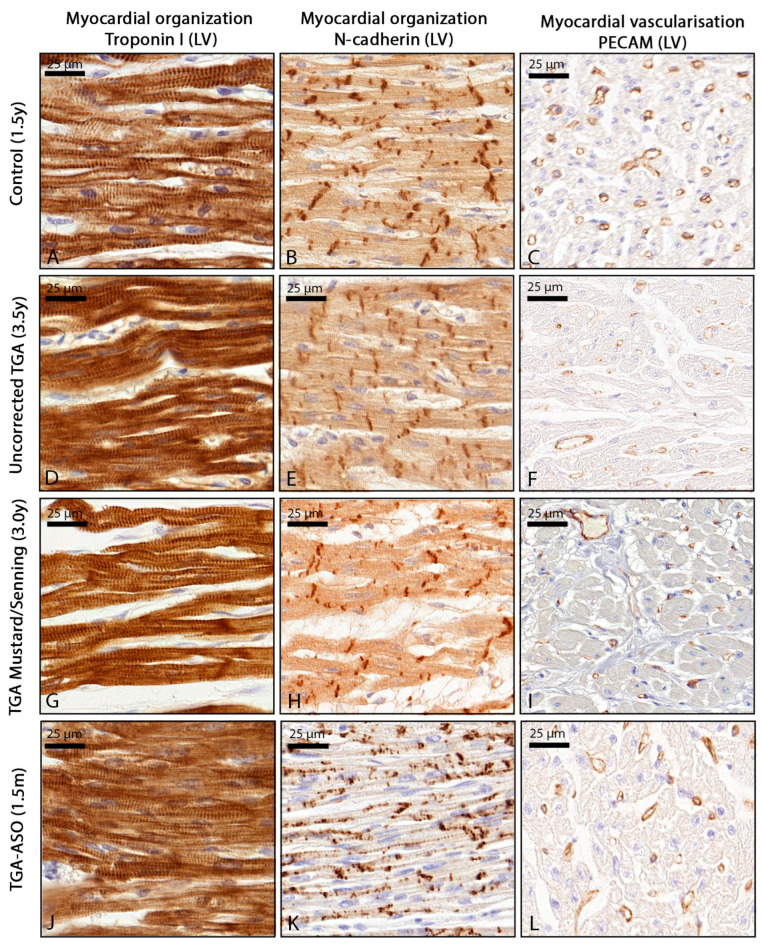
Myocardial organization and vascularization in control, uncorrected TGA, TGA-Mustard/Senning, and TGA-ASO specimens. Control; well-organized cardiomyocyte network (**A**) with expression of NCAD at the intercalated disk (**B**) and homogeneous distribution of capillaries (**C**). Uncorrected TGA; Well organized network of cardiomyocytes (**D**), slightly increased expression of NCAD, mostly located at the intercalated disk (**E**), and less homogeneous capillary distribution (**F**). TGA-Mustard/Senning; increased amount of extracellular matrix (**G**), normal distribution of NCAD at the intercalated disk (**H**), and normal capillary distribution (**I**) TGA-ASO; cardiomyocytes are well aligned (**J**) with increased NCAD distribution at the intercalated disk and lateral borders of the cardiomyocyte (**K**) and decreased amount of capillary distribution (**L**). Abbreviations: ASO, arterial switch operation; d, days; m, months; TGA, transposition of the great arteries; y = years.

Control hearts showed that in both the left and right ventricle, cardiomyocytes were well aligned with a proper banding pattern and a homogeneous distribution of capillaries and cardiomyocytes (Figure 5A–C). Within the uncorrected TGA group, cardiomyocytes were well organized in both systemic RV and subpulmonary LV, although NCAD expression was slightly increased at the gap junctions, and capillary distribution was less homogeneous compared to the control myocardium (Figure 5D–F). In TGA-Mustard/Senning hearts, an increased amount of extracellular matrix was found with disturbed alignment of the cardiomyocytes in both subpulmonary LV and systemic RV (Figure 5G), corresponding with fibrosis areas. In areas with an increased amount of fibrosis, a reduced amount of capillaries was found (Figure 5I). In TGA-ASO specimens, all pediatric specimens showed a normal myocardial structure and capillary distribution (Figure 5J); however, NCAD expression was seen at the intercalated disk and lateral borders of the cardiomyocyte, mostly in specimens younger than 1.5 years (Figure 5K). In the TGA-ASO specimen the amount of capillaries appeared slightly reduced compared to the control (Figure 5L). 

### 3.4. Innervation Post-ASO

**Figure 6:**
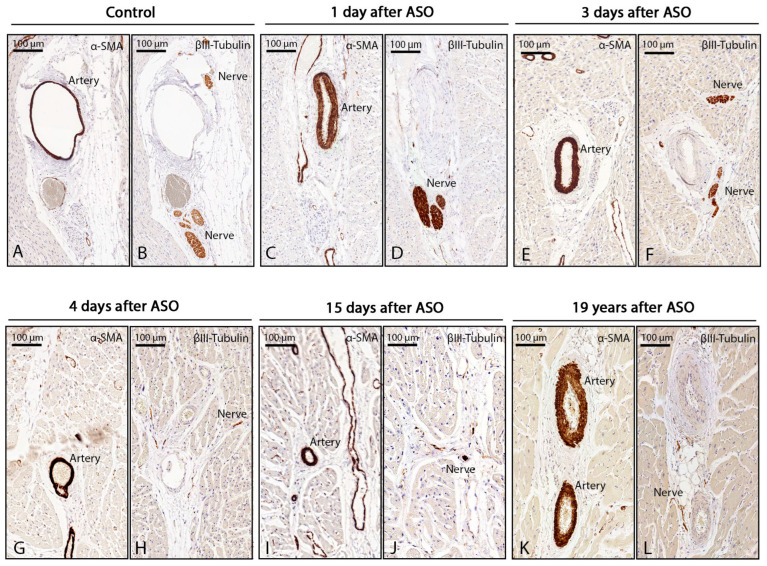
Myocardial innervation in uncorrected TGA and TGA-ASO. Myocardial innervation in uncorrected TGA and after ASO. Innervation post-ASO was evaluated in carefully matched sections in areas with cross-sectioned coronary vessels and compared between controls and TGA-ASO specimens. Visualization of cross-sectioned arteries and veins was performed by α-SMA staining (**A**,**C**,**E**,**G**,**I**,**K**). Innervation staining: (**B**) βIII-tubulin-positive nerves around coronary arteries and veins in uncorrected TGA. (**D**); postmortem hearts that survived 1 day after ASO showed βIII-tubulin-positive nerves, the uptake was comparable to the control specimen. (**F,H,J,L**); reduced uptake of βIII-tubulin around cross-sectioned coronary vessels after 3 days, 4 days, 15 days, and 19 years after ASO. Abbreviations: ASO, arterial switch operation; TGA, transposition of the great arteries; α-SMA, α smooth muscle actin.

**Figure 7:**
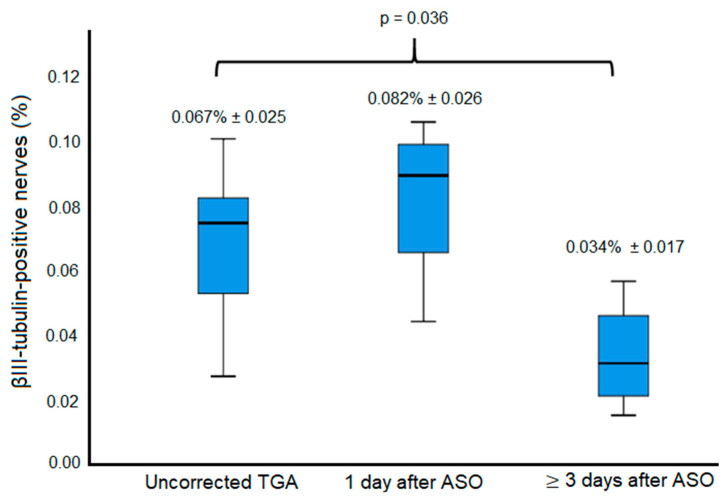
Comparison of βIII-tubulin-positive nerves in TGA-ASO and uncorrected TGA. Boxplot: the mean amount of βIII-tubulin-positive nerves between the control group (uncorrected TGA) (n = 8), TGA 1 day after ASO (n = 4), and TGA ≥ 3 days after ASO (n = 4). Abbreviations: ASO, arterial switch operation; TGA, transposition of the great arteries.

On the histological assessment of myocardial innervation in TGA-ASO specimens, we found that in the specimens that died one day after ASO, the amount of βIII-tubulin positive nerves was not significantly different compared to uncorrected TGA specimens (0.082% ± 0.026 vs. 0.067% ± 0.025, respectively, *p* = 0.451) (Figure 6B,D). However, in specimens of children who survived at least 3 days after ASO (Figure 6F,H,J,L), the amount of βIII-tubulin-positive nerves was significantly reduced compared to uncorrected TGA specimens (0.034% ± 0.017 vs. 0.067% ± 0.025, *p* = 0.036) (Figure 7)

### 3.5. Literature Review on Myocardial Fibrosis in TGA

To correlate our findings with the literature, we systematically reviewed current literature related to TGA with respect to the subjects of our study. The literature search on myocardial fibrosis identified a total of 321 studies. After abstract screening and full text assessment, the majority of studies were excluded as they did not focus on TGA patients (See Figure S2). After careful selection, the following fibrosis studies could be included; 1. CMR imaging studies after Mustard/Senning correction (n = 8) or ASO (n = 3); 2. Histological studies (n = 3) in TGA-Mustard/Senning patients. These papers and their main outcomes are summarized in Table 3. No studies on cardiac fibrosis in uncorrected TGA hearts were found.

#### 3.5.1. CMR Imaging Studies after Mustard/Senning Correction

Eight CMR studies (Table 3) have been performed in TGA patients after Mustard/Senning correction, focusing on the detection of fibrosis using late gadolinium enhancement (LGE) or measuring T1 times and ECV. LGE CMR has become the reference standard for non-invasive imaging of myocardial scar and focal fibrosis. Several LGE CMR studies that evaluated more than 30 TGA patients after Mustard/Senning, at a median age of 26 years, reported RV LGE in up to 61% of the patients, with various patterns. Other studies using T1 mapping methods reported higher ECV in both subpulmonary LV and systemic RV [5,10], suggesting the presence of diffuse interstitial fibrosis. 

#### 3.5.2. Histological Studies after Mustard/Senning Correction

Only three histological studies in TGA-Mustard/Senning were identified [11,12,13] in which diffuse interstitial fibrosis was frequently found in the systemic RV and, to a lesser extent, in the subpulmonary LV [11,13]. However, these results are based on a limited number of specimens (two studies, each with one heart after heart transplantation) or data based on endocardial biopsies only. 

#### 3.5.3. CMR Imaging Studies after TGA-ASO

In the TGA-ASO cohort, three CMR studies focusing on cardiac fibrosis have been performed. Most studies did not find LGE in the LV or RV, whereas one study reported LGE in either the inferior or superior septal-free wall, which was, in the majority, a small focal enhancement. Although higher ECV values with CMR were found post-ASO in both LV and RV compared to controls, this was not associated with any specific area (Table 3). No histological studies on myocardial fibrosis post-ASO were found in our query. 

### 3.6. Literature Review on Cardiac Innervation in TGA-ASO

The literature search on innervation post-arterial switch identified a total of 56 papers. After abstract screening and full text assessment, 53 papers were excluded. Most of these publications were excluded because no TGA-ASO patients were included or the focus was not on cardiac innervation. We included three studies that reported on cardiac innervation after the arterial switch operation (see PRISMA flowchart in the online supplement). These studies reported altered cardiac sympathetic innervation, measured by [*^11^C*]meta-hydroxyephedrine uptake in childhood and also during adulthood, indicating impaired myocardial innervation after ASO. Regional uptake was seen more frequently found at the left ventricle anterior site compared to the lateral site. No histological studies on cardiac innervation post-ASO were found. The included studies and the main results are summarized in Table 4.

## 4. Discussion

In this study, we systematically described the myocardial alterations in the full spectrum of TGA in uncorrected specimens as well as after surgical correction (Mustard/Senning or ASO). The key finding of our study is that interstitial myocardial fibrosis is already present in young uncorrected TGA hearts in both the systemic RV and subpulmonary LV. More fibrosis was observed in older uncorrected TGA patients. Furthermore, after TGA-Mustard/Senning correction, specimens showed various patterns of mild and severe interstitial fibrosis, which was remarkably more pronounced in the subpulmonary LV compared to the systemic RV. In TGA-ASO specimens, we found that the amount of βIII-tubulin-positive nerves was significantly decreased from three days after ASO, which may be related to denervation during the ASO. 

### 4.1. Early Interstitial Myocardial Fibrosis in TGA

In the current study, the amount of interstitial fibrosis was increased in TGA neonates compared to the control group, suggesting that altered hemodynamics and saturations in TGA may induce fibroblast activation, which could already start in utero. In utero remodeling in the setting of congenital heart disease is supported by a study by Zwanenburg et al. [23] that also demonstrates myocardial remodeling and fibrosis in fetal specimens with various degrees of aortic stenosis. Data on remodeling in fetuses with TGA is currently not available. The concept presented in the current study might serve as a base for future studies in fetal TGA specimens. To our knowledge, literature on the proportion of cardiac collagen in the normal ventricular myocardium is only available in adults, in which mean or median proportions varied between 6.5% and 15.2% [8,9,24]. Miles et al. [24] reported in structurally normal hearts the following percentages of fibrosis at a median age of 32.1 years: 15.2% in the RV, 9.5% in LV, and 8.6% in IVS. These values, however, also include the percentage of collagen in the epicardial and endocardial surface, that stain highly for collagen markers, which was not included in our study and made it questionable whether these values could be used as a reference for myocardial fibrosis. Furthermore, Glashan et al. [9], using the same method as employed in the current study, reported a reference value of 6.5% (IQR 4.9–9.3) at a median age of 65 years. Differences in reported fibrosis percentages could be explained by different quantification techniques and methods. In our analysis, we used a semi-automated quantification approach that may underestimate the amount of myocardial collagen area compared to stereological light microscopy techniques [25]. In addition, tissue thickness may play a role in collagen quantification, as a positive relationship was found between automated collagen quantification and increasing section thickness [25]. Finally, variation in reported fibrosis patterns in “normal” postmortem hearts might also be non-physiological, as in many cases underlying pathology contributing to demise might be present, which may also affect the cardiovascular status, such as pulmonary hypertension [26]. 

### 4.2. Myocardial Fibrosis after Surgical Correction

TGA-Mustard/Senning postmortem specimens showed interstitial fibrosis, the amount varying from mild to severe. This finding is in line with a previous histological study in alive TGA-Mustard/Senning patients in which endomyocardial biopsies were obtained during cardiac catheterization at a mean age of 16.9 years and interstitial fibrosis in both the systematic RV as well as in the subpulmonary LV was found [13]. However, in contrast to our study, these findings were focused only on the subendocardial site, and biopsies were stained with a general marker for myocardial structure (Hematoxylin and Eosin), not with a specific collagen marker for fibrosis. Interstitial fibrosis in these biopsies was not associated with systolic ventricular dysfunction at a young age, but an effect on the occurrence of later systolic or diastolic dysfunction later in life cannot be excluded [27]. The finding of diffuse interstitial fibrosis in the systemic RV ventricle is not surprising due to the longstanding pressure load on the morphological RV initiating adverse remodeling. Remarkably, and in accordance with the findings of Gorenflo et al. in living patients mentioned above [13], we also demonstrated an increased amount of diffuse interstitial fibrosis in the subpulmonary LV. The mechanism of this phenomenon is still unclear. 

In our study, analyses of the presence of fibrosis in TGA-ASO were limited and performed in only two specimens due to the limited availability of specimens surviving at least two weeks after ASO. These two specimens died 15 days (age 1.5 months) and 19 years (age 19 years) after ASO. The specimen that died 19 years after ASO remarkably showed a severe amount of patchy fibrosis in the IVS and LV and diffuse interstitial fibrosis in the RV. Detailed clinical data were not available for clinical correlation, although no history of LV disease had been reported. Previous clinical studies with the inclusion of TGA-ASO patients and speckle-tracking strain imaging reported altered ventricular function in TGA patients. Klitsie et al. [28] reported that biventricular function decreased directly postoperatively. Although the LV function recovered, the right ventricular systolic and diastolic function was still impaired in the first year compared to the control group. These results also suggest that myocardial remodeling is already present in young TGA patients. This is also supported by the findings of Di Salvo et al. [29], who report that in 62 asymptomatic TGA-ASO patients (mean age 8.5 years), LV longitudinal strain was significantly lower compared to healthy controls and that the lower strain values were significantly correlated with later age at surgery and could be explained by remodeling of the myocardium before ASO. The clinical impact of diffuse myocardial fibrosis on long-term outcomes in TGA-ASO is still unknown. The incidence of ventricular arrhythmias in the TGA-ASO cohort is low so far, although higher than in the general population [30]. However, the TGA-ASO population is still relatively young, and longer follow-up is necessary to correlate findings of an increased amount of fibrosis to long-term clinical outcomes. 

### 4.3. Myocardial Innervation Post-ASO

The decreased number of βIII-tubulin-positive nerves that were found in specimens who survived at least 3 days after ASO could implicate that denervation starts shortly post-ASO. From a mechanistic point of view, this seems plausible as the cardiac plexus located at the myocardial outflow tract is acutely dissected during the ASO. This is in line with findings reported by Kondo et al. [20], in which innervation, measured by MIBG uptake, was assessed before and after ASO. Patients after ASO showed a complete absence of uptake, indicating denervation, within one month after ASO. This recovered to various degrees late after ASO. In our study, the cardiac specimen of the patient who survived 19 years after ASO still showed reduced βIII-tubulin staining, suggesting that myocardial innervation may still be impaired late after ASO in some patients. These findings correspond to data known in patients after cardiac transplantation, who also show early denervation of the donor’s heart, with various amounts of reinnervation in further follow-up [31,32]. 

### 4.4. Limitations

Several limitations should be acknowledged. First, this study is performed in selected postmortem specimens from the Leiden Collection of Congenital Malformations (that is part of the LUMC Biobank Congenital Heart Disease), and therefore per definition, in patients with a detrimental outcome. Findings should be, therefore, interpreted with caution as they may not be representative of myocardial structure and organization in patients with a more favorable outcome. Histological findings described, however, do correlate with findings in clinical studies reported to date, potentially representing patients at higher risk of complications/events. As TGA is a rare defect and short-term outcome has dramatically improved, the number of specimens in this study was dictated by the availability of postmortem TGA specimens in our biobank that met our inclusion criteria. Nevertheless, to the best of our knowledge, this is the largest histological study reported in postmortem TGA specimens to date. Although our results indicate a potential development of myocardial fibrosis already in utero, we did not examine fetal specimens in the current study. Most available specimens were from previous decades and had been stored for a significant time in conservative solutions. Some specimens did not retain antigen for myocardial structure (NCAD) or vascularization (PECAM), possibly caused by the long storage duration, and were excluded from sub-analysis. Only a limited number of TGA-ASO specimens with the survival of at least five days after the correction was available; therefore, this group was underrepresented to draw valid conclusions with regard to fibrosis patterns over time. Nevertheless, innervation patterns could be studied, and this group of ASO hearts represents the first to be described in histological detail to the best of our knowledge. 

## 5. Conclusions

Histological assessment in a selected group of postmortem TGA hearts showed diffuse interstitial myocardial fibrosis in uncorrected TGA newborns in the systemic RV as well as in the subpulmonary LV. This suggests that myocardial remodeling in TGA may already occur at the fetal stage. Furthermore, in TGA hearts after the Mustard/Senning operation, an increased amount of diffuse interstitial fibrosis was found in both systemic RV and, remarkably, even more so in the subpulmonary LV. Specimens of TGA patients who survived at least three days after ASO showed a decreased amount of autonomic nerves, which may be caused by (partial) denervation during the ASO. Although data from the current study support a careful follow-up of TGA patients, these data should also be interpreted with caution as they are derived from a selected group of patients with detrimental outcomes. Long-term follow-up will likely shed light on the potential clinical implications of increased fibrosis, disturbed innervation patterns, and myocardial disorganization in the clinical group of TGA patients.

## Figures and Tables

**Table 1:** Characteristics of the postmortem hearts.

	Uncorrected TGA (n = 8)	TGA-Mustard/Senning (n = 6)	TGA-ASO (n = 8)
Age (years), median	3.9	3.5	3.7
Age category, n (%)			
1 day	1 (13%)	0 (0%)	1 (13%)
1–3.5 months	2 (25%)	0 (0%)	3 (38%)
1–5 years	4 (50%)	5 (83%)	3 (38%)
19–33 years	1 (13%)	1 (17%)	1 (13%)
Sex (male), n (%)	6 (75%)	4 (67%)	7 (88%)
TGA subtype, n (%)			
TGA-IVS	3 (38%)	3 (50%)	2 (25%)
TGA-VSD	3 (38%)	1 (17%)	4 (50%)
TB-DORV	2 (25%)	2 (33%)	2 (25%)
Shunts			
Foramen ovale, n (%)			
Open	8 (100%)	-	0 (0%)
Closed after ASO	–	-	8 (100%)
Closed	0 (0%)	-	0 (0%)
Ductus arteriosus, n (%)			
Open	4 (50%)	0 (0%)	0 (0%)
Closed (after surgery)	0 (0%)	1 (17%)	4 (50%)
Unknown	4 (50%)	5 (83%)	4 (50%)
Cause of death, n (%)			
Cardiac death	4 (50%)	5 (83%)	7 (88%)
Other	2 (25%)	1 (17%)	1 (13%)
Unknown	2 (25%)	0 (0%)	0 (0%)
Procedures, n (%)			
Rashkind	2 (25%)	3 (50%)	0 (0%)
Blalock-Hanlon	1 (13%)	1 (17%)	1 (13%)
Time between surgical correction and death, category, n (%)			
1–4 days	-	0 (0%)	6 (75%)
15 days		-	1 (13%)
6 months	-	1 (17%)	-
1–3 years	-	4 (67%)	-
>19 years	-	1 (17%)	1 (13%)

Characteristics of the postmortem specimens; uncorrected TGA, TGA-Mustard/Senning, and TGA-ASO. Abbreviations: ASO, arterial switch operation; DORV, double outlet right ventricle; IVS, intact ventricular septum; TB, taussig bing anomaly; TGA, transposition of the great arteries; VSD, ventricular septum defect.

**Table 2:** Quantification of fibrosis in control, uncorrected TGA, TGA-Mustard/Senning, and TGA-ASO.

Control	Diagnosis	Age		Systemic LV	Subpulmonary RV	IVS	Mean ^a^
#1	No-CHD	1 day		4.2%	4.9%	5.4%	4.8%
#2	No-CHD	1 month		5.2%	6.4%	6.1%	5.9%
#3	No-CHD	1.5 years		6.7%	7.1%	6.5%	6.8%
			*mean* ^b^	5.4%	6.1%	6.0%	
Uncorrected TGA	Diagnosis	Age		Subpulmonary LV	Systemic RV	IVS	Mean ^a^
#4	TGA-IVS	1 day		5.2%	6.1%	8.7%	6.7%
#5	TB-DORV	1 month		6.6%	8.9%	8.4%	8.0%
#6	TB-DORV	1.5 months		13.8%	13.4%	8.6%	11.9%
#7	TGA-VSD	2.5 years		5.8%	9.7%	7.1%	7.5%
#8	TGA-IVS	2.5 years		17.9%	16.2%	22.2%	18.8%
#9	TGA-VSD	3.5 years		9.2%	10.5%	10.7%	10.1%
#10	TGA-IVS	5.5 years		6.2%	8.9%	8.0%	7.7%
#11	TGA-VSD	20 years		7.8%	8.0%	19.8%	11.9%
			*mean* ^b^	9.1%	10.2%	11.7%	
TGA-Mustard/Senning	Diagnosis	Age		Subpulmonary LV	Systemic RV	IVS	Mean ^a^
#12	TGA-VSD	1 year		36.7%	21.8%	23.5%	27.3%
#13	TGA-IVS	1.8 years		11.9%	16.1%	10.7%	12.9%
#14	TGA-IVS	2.5 years		14.9%	15.1%	17.1%	15.7%
#15	TB-DORV	3.5 years		26.2%	17.5%	20.8%	21.5%
#16	TGA-IVS	5 years		21.3%	16.5%	21.1%	19.6%
#17	TB-DORV	33 years		23.4%	17.7%	24.5%	21.9%
			*mean* ^b^	22.4%	17.5%	19.6%	
TGA-ASO	Diagnosis	Age		Systemic LV	Subpulmonary RV	IVS	Mean ^a^
#18	TGA-IVS	1 day		-	-	-	-
#19	TGA-VSD	1 month		-	-	-	-
#20	TGA-IVS	1.5 months		6.8%	7.4%	6.9%	7.0%
#21	TGA-VSD	3.5 months		-	-	-	-
#22	TGA-VSD	2 years		-	-	-	-
#23	TGA-VSD	3.5 years		-	-	-	-
#24	TB-DORV	4.5 years		-	-	-	-
#25	TGA-IVS	19 years		14.8%	18.4%	22.0%	18.4%
			*mean* ^b^	10.8%	12.9%	14.5%	

Quantification of fibrosis in control, Uncorrected TGA, TGA-Mustard/Senning, and TGA-ASO. Of the TGA group, only hearts from patients that survived at least 15 days after ASO were included for fibrosis quantification. ^a^ Mean amount of fibrosis in left ventricle, right ventricle, and interventricular septum; ^b^ Mean amount of fibrosis in each subgroup. Abbreviations: ASO, arterial switch operation; CHD, congenital heart defect; DORV, double outlet right ventricle; IVS, intact ventricular septum; LV, left ventricle; RV, right ventricle; TB, taussig bing anomaly; TGA, transposition of the great arteries; VSD, ventricular septum defect.

**Table 3:** Overview of fibrosis studies.

Author and Year	Surgical Correction	(n)	Age	Controls (n)	Age	Examination	Protocol	Main Findings/Conclusions
Broberg et al. 2018 [5]	Mustard/Senning	53	Mean 34.6 years	22	Mean 40.2 years	CMR	T1 mapping and ECV before and after gadolineum	Significantly higher ECV for the systemic RV (28.7 ± 4.4%) compared to controls (26.1 ± 2.8%, *p* = 0.0104).
Cheung et al. 2020 [10]	Mustard/Senning	31	Mean 33.3 years	-	-	CMR	T1 mapping and ECV before and after gadolineum	Patients had significantly greater RV and LV native T1 times and ECV values (all *p* < 0.001).
Ladouceur et al. 2009 [11]	Senning	1	27.1 years	-	-	CMR and microscopic	LGE before transplantation and RV biopsies post-transplantation	LGE in RV inferior wall and septum. Dense and contiguous fibrosis (25%) in RV inferior wall and diffuse interstitial fibrosis at many sites.
Rydman et al. 2015 [12]	Mustard/Senning	55	Mean 27.0 years	-	-	CMR and microscopic	CMR and histological assessment	RV LGE was present in 31 patients (56%). Histological assessment post-transplantation (n = 1): focal fibrosis in RV and diffuse fibrosis in the septum, and no fibrosis in LV.
Gorenflo et al. 2003 [13]	Mustard/Senning	12	Median 16.9 years	-	-	Microscopic	Endomyocardial biopsies from LV and RV	All systemic RV biopsies showed interstitial fibrosis, and 6/12 patients showed fibrous and fatty degeneration of the subpulmonary LV.
Babu-Narayan et al. 2005 [14]	Mustard/Senning	36	Mean 27.0 years	-	-	CMR	LGE	Late gadolineum RV enhancement was seen in 61% of patients with various patterns. The extent of LGE correlated with age.
Plymen et al. 2013 [15]	Mustard/Senning	14	Median 33.7 years	14	Mean 32.0 years	CMR	T1 mapping and ECV before and after gadolineum	No transmural LGE was observed. Septal ECV was significantly higher in patients than in controls.
Ladouceur et al. 2018 [16]	Mustard/Senning	48	Median 32 years	-	-	CMR	LGE	LGE was present in RV in 17 patients (35%), mainly at basal segment (54%).
Shehu et al. 2018 [17]	Mustard/Senning	10	Mean 36.8 years	-	-	CMR	T1 mapping and ECV before and after gadolineum	ECV of the inferior and lateral wall of the LV was significantly increased compared to the RV.
Grotenhuis et al. 2018 [6]	ASO	30	Mean 15.4 years	28	Mean 14.1 years	CMR	ECV, native T1 times and LGE	Native T1 times were significantly higher in the entire LV and septum. No myocardial scarring.
Shepard et al. 2016 [18]	ASO	220	Median 15.4 years	-	-	CMR	Late enhancement	LGE was found in either the inferior or the superior septal-free wall (most had a nonischemic pattern).
Suther et al. 2018 [19]	ASO	30	Mean 11.7 years	15	Mean 22.4 years	CMR	ECV, pre- and post-contrast T1 mapping and LGE	Increased ECV in all coronary territories.

Overview of fibrosis studies in TGA. Abbreviations; ASO, arterial switch operation; CMR, cardiac magnetic resonance; ECV, extracellular volume; LGE, late gadolinium enhancement; LV, left ventricle; TGA, transposition of the great arteries; RV, right ventricle.

**Table 4:** Overview innervation studies after arterial switch operation.

Author and Year	ASO (n)	Age	Controls (n)	Age	Examination	Main Findings/Conclusions
Kondo et al. 1998 [20]	51	Mean 4,8 years	4	Mean 3.0 months	MIBG scintigraphy	Early after ASO (<1 month) complete absence of MIBG uptake (n = 4) was seen. Although, late after ASO (15 months–10.1 years), there were various degrees of MIBG uptake
Kuehn et al. 2014 [21]	9	Mean 20.8 years	9	Mean 22.1 years	PET: epinephrine retention LV	Signs of reinnervation in most of the patients after ASO and only 1 patient in each group showed complete denervation with epinephrine retention <7%/min
Possner et al. 2020 [22]	12	Mean 22.5 years	10	Mean 22.0 years	PET: meta-hydroxyephedrine uptake	Global meta-hydroxyephedrine uptake was significantly lower in patients compared to controls

Overview of innervation studies in TGA-ASO. Abbreviations; ASO, arterial switch operation; LV, left ventricle; MIBG, metaiodobenzylguanidine; PET, positron emission tomography; TGA, transposition of the great arteries.

## Data Availability

Data are available upon reasonable request.

## Supplementary Materials

The following supporting information can be downloaded at: https://www.mdpi.com/article/10.3390/jcdd10040180/s1, Figure S1: Fibrosis quantification steps; Figure S2: PRISMA flow diagram, Table S1: List of antibodies; Text S1: search strategy myocardial fibrosis in TGA; Text S2: search strategy myocardial innervation post-ASO.
