# Supplementary material for: Cardiac Fibrosis and Innervation State in Uncorrected and Corrected Transposition of the Great Arteries: A Postmortem Histological Analysis and Systematic Review
Source: J Cardiovasc Dev Dis. 2023 Apr 20;10(4):180. doi: 10.3390/jcdd10040180 (PMC10143292; doi:10.3390/jcdd10040180)
Supplement: Supplementary file 1 [file jcdd-10-00180-s001.zip › jcdd-2281779-supplementary.pdf]

Supplement:

Supplemental Figure S1: Fibrosis quantification steps

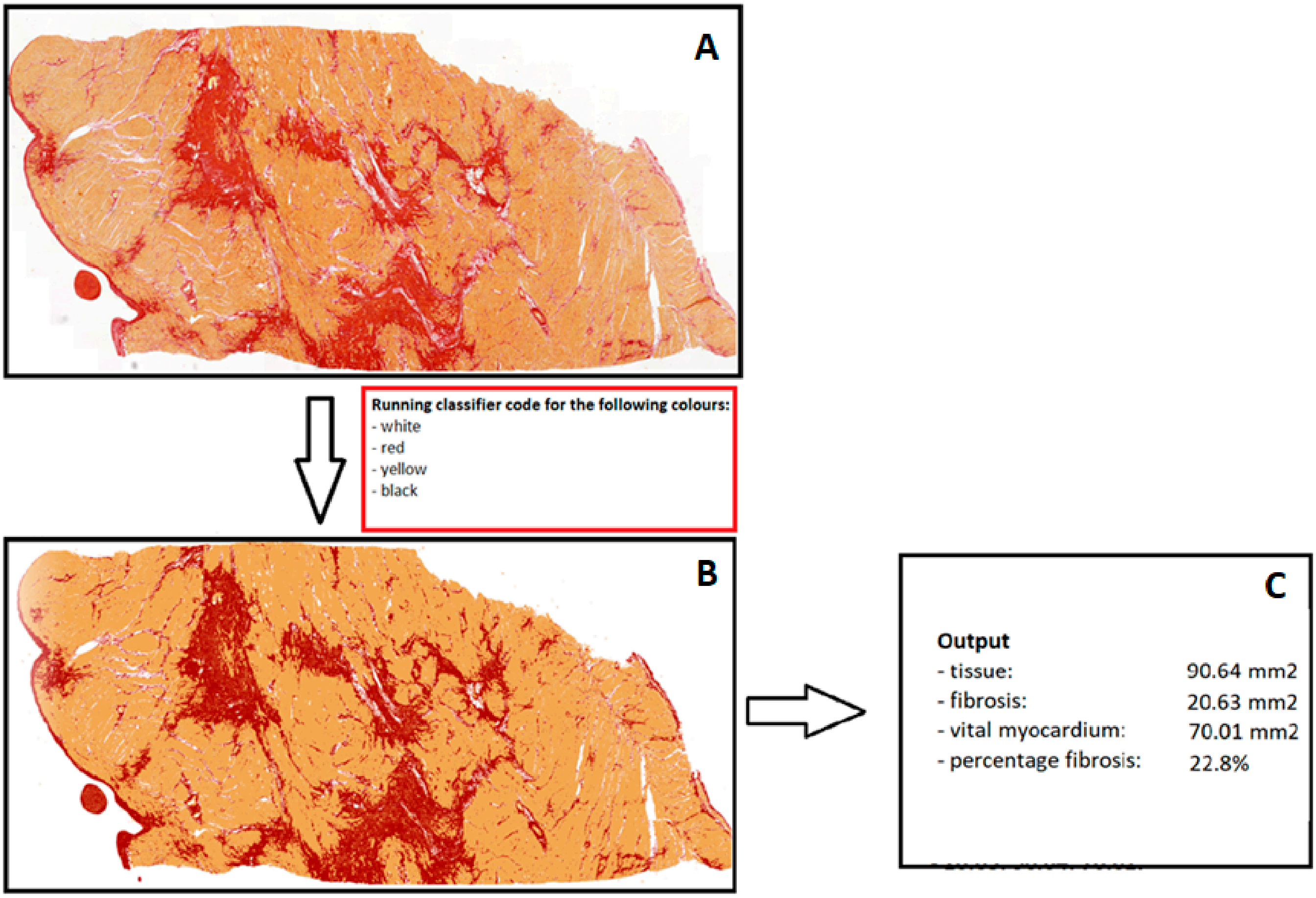

Supplemental Figure S1: Fibrosis quantification steps with Custom software (Python, 2.7). Panel A: Image of Picrosirius Red staining of the myocardium. Wall thickness for each biopsy was measured and each pixel in the image was classified as red (collagen), yellow (myocardium), black (nuclei) or white (non-staining tissue) (Panel B). The amount of collagen was calculated as percentage of the total tissue area by dividing the number of red pixels by the sum of red, black, and yellow pixels (Panel C)

Supplemental Figure S2: PRISMA flow diagram; search on myocardial fibrosis in TGA and cardiac innervation post ASO

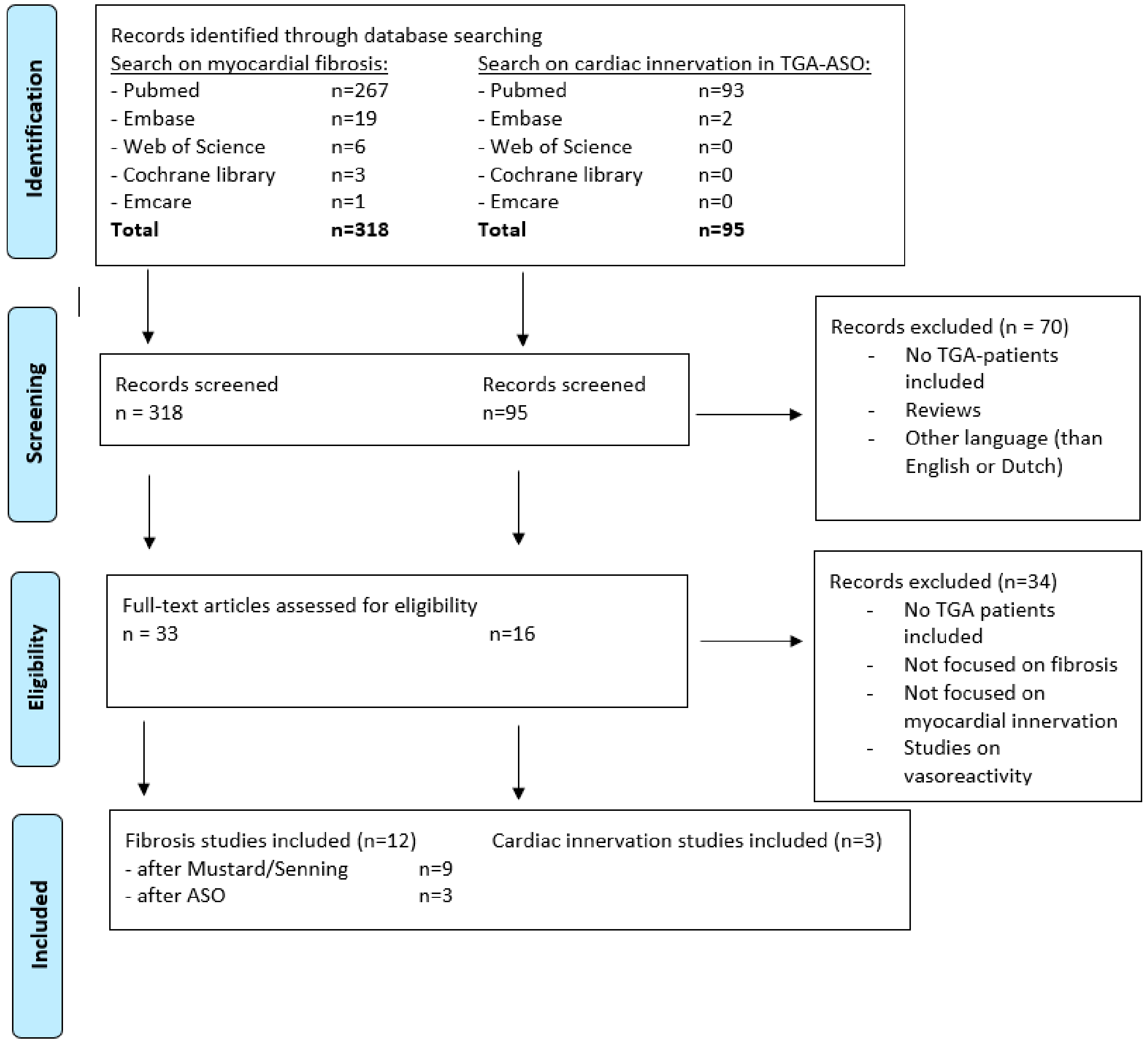

Supplemental Figure S2. PRISMA flow diagram; Literature search on myocardial fibrosis in TGA and cardiac innervation post-ASO. Abbreviations; ASO, arterial switch operation; TGA, transposition of the great arteries;

Supplemental Table S1. List of antibodies

Primary Antibodies	Indication	Species	Clonality	Dilution	Source
Cardiac troponin-I	Myocardial structure	Rabbit	Polyclonal	1:1000	Abcam; AB47003
TUBB3	Cardiac innervation	Rabbit	Polyclonal	1:8000	Sigma-Aldrich; T3952
N-cadherin *	Adherence junctions	Mouse	Monoclonal	1:200	Sigma-Aldrich; C3865
PECAM1 *	Endothelial cells	Rabbit	Polyclonal	1:1000	Santa Cruz; sc1506R

Supplemental Table S1. List of primary antibodies. Abbreviations; TUBB3, βIII-tubulin; PECAM-1, platelet endothelial cell adhesion molecule-1.

Search strategy myocardial fibrosis in TGA:

PubMed
http://www.ncbi.nlm.nih.gov/pubmed?otool=leiden

((("Transposition of Great Vessels"[Mesh] OR "transposition of the great arteries"[tw] OR "transposition of the great artery"[tw] OR "transposition of great arteries"[tw] OR "transposition of great artery"[tw] OR "transposition of the great vessels"[tw] OR "transposition of great vessels"[tw] OR "transposition of great vessel"[tw] OR "great vessels transposition"[tw] OR "great arteries transposition"[tw] OR "congenitally corrected transposition"[tw] OR "congenitally corrected transpositions"[tw] OR "double outlet right ventricle"[tw] OR "double outlet right ventricles"[tw] OR "taussig bing anomaly"[tw] OR (("transposition"[tw] OR "transpositions"[tw] OR "transpos*"[tw] OR ("TGA"[tw] NOT "transient global amnesia"[tw])) AND ("great vessel"[tw] OR "great vessels"[tw] OR "great arteries"[tw] OR "great artery"[tw] OR "large vessel"[tw] OR "large vessels"[tw] OR "large arteries"[tw] OR "large artery"[tw] OR "Aorta"[mesh] OR "aorta"[tw] OR "aortas"[tw] OR "aortic"[tw])) OR "Arterial Switch Operation"[Mesh] OR "atrial switch"[tw] OR "arterial switch"[tw] OR "atrial switch*"[tw] OR "arterial switch*"[tw] OR "double switch operation"[tw] OR "double switch procedure"[tw] OR "double switch technique"[tw] OR "double switch operations"[tw] OR "double switch procedures"[tw] OR "double switch"[tw] OR "jatene operation"[tw] OR "jatene procedure"[tw] OR "jatene technique"[tw] OR "mustard operation"[tw] OR "mustard procedure"[tw] OR "mustard repair"[tw] OR "rastelli operation"[tw] OR "rastelli procedure"[tw] OR "rastelli repair"[tw] OR "rastelli technique"[tw] OR "senning operation"[tw] OR "senning procedure"[tw] OR "jatene"[tw] OR "mustard child*"[tw] OR "mustard patient*"[tw] OR "rastelli"[tw] OR "senning"[tw]) AND ("Fibrosis"[Mesh:noexp] OR "fibrosis"[tw] OR "fibroses"[tw] OR "fibrotic"[tw] OR "fibros*"[tw] OR "fibrot*"[tw] OR "fibrous"[tw] OR "Fibroblasts"[mesh:noexp] OR "fibroblast"[tw] OR "fibroblasts"[tw] OR "Myofibroblasts"[mesh] OR "myofibroblast"[tw] OR "myofibroblasts"[tw] OR "fibroblast*"[tw] OR "fibrocellular"[tw] OR "fibrocell*"[tw] OR "fibrocollagen*"[tw] OR "fibrocollagenous"[tw] OR "myocardial remodelling"[tw] OR "myocardial remodeling"[tw] OR "myocardium remodelling"[tw] OR "myocardium remodeling"[tw] OR "myocardial remodel*"[tw] OR "myocardium remodel*"[tw] OR "Ventricular Remodeling"[Mesh] OR "Ventricular Remodelling"[tw] OR "Ventricular Remodeling"[tw] OR "Ventricle Remodelling"[tw] OR "Ventricle Remodeling"[tw] OR "Ventricular Remodel*"[tw] OR "Ventricle Remodel*"[tw] OR "cardiac remodelling"[tw] OR "cardiac remodeling"[tw] OR "cardiac remodel*"[tw]) NOT (27885969[uid])) **OR** (("Arterial Switch Operation"[Mesh] OR "atrial switch"[tw] OR "arterial switch"[tw] OR "atrial switch*"[tw] OR "arterial switch*"[tw] OR "double switch operation"[tw] OR "double switch procedure"[tw] OR "double switch technique"[tw] OR "double switch operations"[tw] OR "double switch procedures"[tw] OR "double switch"[tw] OR "jatene operation"[tw] OR "jatene procedure"[tw] OR "jatene technique"[tw] OR "mustard operation"[tw] OR "mustard procedure"[tw] OR "mustard repair"[tw] OR "rastelli operation"[tw] OR "rastelli procedure"[tw] OR "rastelli repair"[tw] OR "rastelli technique"[tw] OR "senning operation"[tw] OR "senning procedure"[tw] OR "jatene"[tw] OR "mustard child*"[tw] OR "mustard patient*"[tw] OR

"rastelli"[tw] OR "senning"[tw]) AND ("Histological Techniques"[Mesh] OR "Histology"[Mesh] OR "histology"[tw] OR "histolog*"[tw] OR "histocyto*"[tw])))

Embase
http://ovidsp.ovid.com/ovidweb.cgi?T=JS&PAGE=main&MODE=ovid&D=oemezd

(((exp *"Great Vessels Transposition"/ OR "transposition of the great arteries".ti,ab OR "transposition of the great artery".ti,ab OR "transposition of great arteries".ti,ab OR "transposition of great artery".ti,ab OR "transposition of the great vessels".ti,ab OR "transposition of great vessels".ti,ab OR "transposition of great vessel".ti,ab OR "great vessels transposition".ti,ab OR "great arteries transposition".ti,ab OR "congenitally corrected transposition".ti,ab OR "congenitally corrected transpositions".ti,ab OR "double outlet right ventricle".ti,ab OR "double outlet right ventricles".ti,ab OR "taussig bing anomaly".ti,ab OR (("transposition".ti,ab OR "transpositions".ti,ab OR "transpos*".ti,ab OR ("TGA".ti,ab NOT "transient global amnesia".ti,ab)) AND ("great vessel".ti,ab OR "great vessels".ti,ab OR "great arteries".ti,ab OR "great artery".ti,ab OR "large vessel".ti,ab OR "large vessels".ti,ab OR "large arteries".ti,ab OR "large artery".ti,ab OR exp *"Aorta"/ OR "aorta".ti,ab OR "aortas".ti,ab OR "aortic".ti,ab OR exp *"great blood vessel"/)) OR exp *"Arterial Switch Operation"/ OR "atrial switch".ti,ab OR "arterial switch".ti,ab OR "atrial switch*".ti,ab OR "arterial switch*".ti,ab OR "double switch operation".ti,ab OR "double switch procedure".ti,ab OR "double switch technique".ti,ab OR "double switch operations".ti,ab OR "double switch procedures".ti,ab OR "double switch".ti,ab OR "jatene operation".ti,ab OR "jatene procedure".ti,ab OR "jatene technique".ti,ab OR "mustard operation".ti,ab OR "mustard procedure".ti,ab OR "mustard repair".ti,ab OR "rastelli operation".ti,ab OR "rastelli procedure".ti,ab OR "rastelli repair".ti,ab OR "rastelli technique".ti,ab OR "senning operation".ti,ab OR "senning procedure".ti,ab OR "jatene".ti,ab OR "mustard child*".ti,ab OR "mustard patient*".ti,ab OR "rastelli".ti,ab OR "senning".ti,ab) AND (*"Fibrosis"/ OR *"heart muscle fibrosis"/ OR "fibrosis".ti,ab OR "fibroses".ti,ab OR "fibrotic".ti,ab OR "fibros*".ti,ab OR "fibrot*".ti,ab OR "fibrous".ti,ab OR *"Fibroblast"/ OR *"Heart Fibroblast"/ OR "fibroblast".ti,ab OR "fibroblasts".ti,ab OR *"Myofibroblast"/ OR "myofibroblast".ti,ab OR "myofibroblasts".ti,ab OR "fibroblast*".ti,ab OR "fibrocellular".ti,ab OR "fibrocell*".ti,ab OR "fibrocollagen*".ti,ab OR "fibrocollagenous".ti,ab OR "myocardial remodelling".ti,ab OR "myocardial remodeling".ti,ab OR "myocardium remodelling".ti,ab OR "myocardium remodeling".ti,ab OR "myocardial remodel*".ti,ab OR "myocardium remodel*".ti,ab OR *"Heart Ventricl Remodeling"/ OR "Ventricular Remodelling".ti,ab OR "Ventricular Remodeling".ti,ab OR "Ventricle Remodelling".ti,ab OR "Ventricle Remodeling".ti,ab OR "Ventricular Remodel*".ti,ab OR "Ventricle Remodel*".ti,ab OR "cardiac remodelling".ti,ab OR "cardiac remodeling".ti,ab OR "cardiac remodel*".ti,ab)) **OR** ((exp *"Arterial Switch Operation"/ OR "atrial switch".ti,ab OR "arterial switch".ti,ab OR "atrial switch*".ti,ab OR "arterial switch*".ti,ab OR "double switch operation".ti,ab OR "double switch procedure".ti,ab OR "double switch technique".ti,ab OR "double switch operations".ti,ab OR "double switch procedures".ti,ab OR "double switch".ti,ab OR "jatene operation".ti,ab OR "jatene procedure".ti,ab OR "jatene technique".ti,ab OR "mustard operation".ti,ab OR "mustard procedure".ti,ab OR "mustard repair".ti,ab OR "rastelli operation".ti,ab OR "rastelli procedure".ti,ab OR "rastelli repair".ti,ab OR "rastelli technique".ti,ab OR "senning operation".ti,ab OR "senning procedure".ti,ab OR "jatene".ti,ab OR "mustard child*".ti,ab OR "mustard patient*".ti,ab OR "rastelli".ti,ab OR "senning".ti,ab) AND (exp *"Histology"/ OR "histology".ti,ab OR "histolog*".ti,ab OR "histocyto*".ti,ab)))

- NOT conference review.pt
- NOT (conference review or conference abstract).pt
- AND (conference abstract).pt

Web of Science
http://isiknowledge.com/wos

(((TI=("Great Vessels Transposition" OR "transposition of the great arteries" OR "transposition of the great artery" OR "transposition of great arteries" OR "transposition of great artery" OR "transposition of the great vessels" OR "transposition of great vessels" OR "transposition of great vessel" OR "great vessels transposition" OR "great arteries transposition" OR "congenitally corrected transposition" OR "congenitally corrected transpositions" OR "double outlet right ventricle" OR "double outlet right ventricles" OR "taussig bing anomaly" OR (("transposition" OR "transpositions" OR "transpos*" OR ("TGA" NOT "transient global amnesia")) AND ("great vessel" OR "great vessels" OR "great arteries" OR "great artery" OR "large vessel" OR "large vessels" OR "large arteries" OR "large artery" OR "Aorta" OR "aorta" OR "aortas" OR "aortic" OR "great blood vessel")) OR "Arterial Switch Operation" OR "atrial switch" OR "arterial switch" OR "atrial switch*" OR "arterial switch*" OR "double switch operation" OR "double switch procedure" OR "double switch technique" OR "double switch operations" OR "double switch procedures" OR "double switch" OR "jatene operation" OR "jatene procedure" OR "jatene technique" OR "mustard operation" OR "mustard procedure" OR "mustard repair" OR "rastelli operation" OR "rastelli procedure" OR "rastelli repair" OR "rastelli technique" OR "senning operation" OR "senning procedure" OR "jatene" OR "mustard child*" OR "mustard patient*" OR "rastelli" OR "senning") OR AK=("Great Vessels Transposition" OR "transposition of the great arteries" OR "transposition of the great artery" OR "transposition of great arteries" OR "transposition of great artery" OR "transposition of the great vessels" OR "transposition of great vessels" OR "transposition of great vessel" OR "great vessels transposition" OR "great arteries transposition" OR "congenitally corrected transposition" OR "congenitally corrected transpositions" OR "double outlet right ventricle" OR "double outlet right ventricles" OR "taussig bing anomaly" OR (("transposition" OR "transpositions" OR "transpos*" OR ("TGA" NOT "transient global amnesia")) AND ("great vessel" OR "great vessels" OR "great arteries" OR "great artery" OR "large vessel" OR "large vessels" OR "large arteries" OR "large artery" OR "Aorta" OR "aorta" OR "aortas" OR "aortic" OR "great blood vessel")) OR "Arterial Switch Operation" OR "atrial switch" OR "arterial switch" OR "atrial switch*" OR "arterial switch*" OR "double switch operation" OR "double switch procedure" OR "double switch technique" OR "double switch operations" OR "double switch procedures" OR "double switch" OR "jatene operation" OR "jatene procedure" OR "jatene technique" OR "mustard operation" OR "mustard procedure" OR "mustard repair" OR "rastelli operation" OR "rastelli procedure" OR "rastelli repair" OR "rastelli technique" OR "senning operation" OR "senning procedure" OR "jatene" OR "mustard child*" OR "mustard patient*" OR "rastelli" OR "senning") OR AB=("Great Vessels Transposition" OR "transposition of the great arteries" OR "transposition of the great artery" OR "transposition of great arteries" OR "transposition of great artery" OR "transposition of the great vessels" OR "transposition of great vessels" OR "transposition of great vessel" OR "great vessels transposition" OR "great arteries transposition" OR "congenitally corrected transposition" OR "congenitally corrected transpositions" OR "double outlet right ventricle" OR "double outlet right ventricles" OR "taussig bing anomaly" OR (("transposition" OR "transpositions" OR "transpos*" OR ("TGA" NOT "transient global amnesia")) AND ("great vessel" OR "great vessels" OR "great arteries" OR "great artery" OR "large vessel" OR "large vessels" OR "large arteries" OR "large artery" OR "Aorta" OR "aorta" OR "aortas" OR "aortic" OR "great blood vessel")) OR "Arterial Switch Operation" OR "atrial switch" OR "arterial switch" OR "atrial switch*" OR "arterial switch*" OR "double switch operation" OR "double switch procedure" OR "double switch technique" OR "double switch operations" OR "double switch procedures" OR "double switch" OR "jatene operation" OR "jatene procedure" OR "jatene technique" OR "mustard operation" OR "mustard procedure" OR "mustard repair" OR "rastelli operation" OR "rastelli procedure" OR "rastelli repair" OR "rastelli technique" OR "senning operation" OR "senning procedure" OR "jatene" OR "mustard child*" OR "mustard patient*" OR "rastelli" OR "senning")) AND (TI=("Fibrosis" OR "heart muscle fibrosis" OR "fibrosis" OR "fibroses" OR "fibrotic" OR "fibros*" OR "fibrot*" OR "fibrous" OR "Fibroblast" OR "Heart Fibroblast" OR "fibroblast" OR "fibroblasts" OR "Myofibroblast" OR "myofibroblast" OR

"myofibroblasts" OR "fibroblast*" OR "fibrocellular" OR "fibrocell*" OR "fibrocollagen*" OR "fibrocollagenous" OR "myocardial remodelling" OR "myocardial remodeling" OR "myocardium remodelling" OR "myocardium remodeling" OR "myocardial remodel*" OR "myocardium remodel*" OR "Heart Ventricl Remodeling" OR "Ventricular Remodelling" OR "Ventricular Remodeling" OR "Ventricle Remodelling" OR "Ventricle Remodeling" OR "Ventricular Remodel*" OR "Ventricle Remodel*" OR "cardiac remodelling" OR "cardiac remodeling" OR "cardiac remodel*") OR AK=("Fibrosis" OR "heart muscle fibrosis" OR "fibrosis" OR "fibroses" OR "fibrotic" OR "fibros*" OR "fibrot*" OR "fibrous" OR "Fibroblast" OR "Heart Fibroblast" OR "fibroblast" OR "fibroblasts" OR "Myofibroblast" OR "myofibroblast" OR "myofibroblasts" OR "fibroblast*" OR "fibrocellular" OR "fibrocell*" OR "fibrocollagen*" OR "fibrocollagenous" OR "myocardial remodelling" OR "myocardial remodeling" OR "myocardium remodelling" OR "myocardium remodeling" OR "myocardial remodel*" OR "myocardium remodel*" OR "Heart Ventricl Remodeling" OR "Ventricular Remodelling" OR "Ventricular Remodeling" OR "Ventricle Remodelling" OR "Ventricle Remodeling" OR "Ventricular Remodel*" OR "Ventricle Remodel*" OR "cardiac remodelling" OR "cardiac remodeling" OR "cardiac remodel*") OR AB=("Fibrosis" OR "heart muscle fibrosis" OR "fibrosis" OR "fibroses" OR "fibrotic" OR "fibros*" OR "fibrot*" OR "fibrous" OR "Fibroblast" OR "Heart Fibroblast" OR "fibroblast" OR "fibroblasts" OR "Myofibroblast" OR "myofibroblast" OR "myofibroblasts" OR "fibroblast*" OR "fibrocellular" OR "fibrocell*" OR "fibrocollagen*" OR "fibrocollagenous" OR "myocardial remodelling" OR "myocardial remodeling" OR "myocardium remodelling" OR "myocardium remodeling" OR "myocardial remodel*" OR "myocardium remodel*" OR "Heart Ventricl Remodeling" OR "Ventricular Remodelling" OR "Ventricular Remodeling" OR "Ventricle Remodelling" OR "Ventricle Remodeling" OR "Ventricular Remodel*" OR "Ventricle Remodel*" OR "cardiac remodelling" OR "cardiac remodeling" OR "cardiac remodel*"))) **OR** ((TI=("Arterial Switch Operation" OR "atrial switch" OR "arterial switch" OR "atrial switch*" OR "arterial switch*" OR "double switch operation" OR "double switch procedure" OR "double switch technique" OR "double switch operations" OR "double switch procedures" OR "double switch" OR "jatene operation" OR "jatene procedure" OR "jatene technique" OR "mustard operation" OR "mustard procedure" OR "mustard repair" OR "rastelli operation" OR "rastelli procedure" OR "rastelli repair" OR "rastelli technique" OR "senning operation" OR "senning procedure" OR "jatene" OR "mustard child*" OR "mustard patient*" OR "rastelli" OR "senning") OR AK=("Arterial Switch Operation" OR "atrial switch" OR "arterial switch" OR "atrial switch*" OR "arterial switch*" OR "double switch operation" OR "double switch procedure" OR "double switch technique" OR "double switch operations" OR "double switch procedures" OR "double switch" OR "jatene operation" OR "jatene procedure" OR "jatene technique" OR "mustard operation" OR "mustard procedure" OR "mustard repair" OR "rastelli operation" OR "rastelli procedure" OR "rastelli repair" OR "rastelli technique" OR "senning operation" OR "senning procedure" OR "jatene" OR "mustard child*" OR "mustard patient*" OR "rastelli" OR "senning") OR AB=("Arterial Switch Operation" OR "atrial switch" OR "arterial switch" OR "atrial switch*" OR "arterial switch*" OR "double switch operation" OR "double switch procedure" OR "double switch technique" OR "double switch operations" OR "double switch procedures" OR "double switch" OR "jatene operation" OR "jatene procedure" OR "jatene technique" OR "mustard operation" OR "mustard procedure" OR "mustard repair" OR "rastelli operation" OR "rastelli procedure" OR "rastelli repair" OR "rastelli technique" OR "senning operation" OR "senning procedure" OR "jatene" OR "mustard child*" OR "mustard patient*" OR "rastelli" OR "senning")) AND (TI=("Histology" OR "histology" OR "histolog*" OR "histocyto*") OR AK=("Histology" OR "histology" OR "histolog*" OR "histocyto*") OR AB=("Histology" OR "histology" OR "histolog*" OR "histocyto*"))))

Cochrane
https://www.cochranelibrary.com/advanced-search/search-manager

((("Great Vessels Transposition" OR "transposition of the great arteries" OR "transposition of the great artery" OR "transposition of great arteries" OR "transposition of great artery" OR "transposition of the great vessels" OR "transposition of great vessels" OR "transposition of great vessel" OR "great vessels transposition" OR "great arteries transposition" OR "congenitally corrected transposition" OR "congenitally corrected transpositions" OR "double outlet right ventricle" OR "double outlet right ventricles" OR "taussig bing anomaly" OR (("transposition" OR "transpositions" OR "transpos*" OR ("TGA" NOT "transient global amnesia")) AND ("great vessel" OR "great vessels" OR "great arteries" OR "great artery" OR "large vessel" OR "large vessels" OR "large arteries" OR "large artery" OR "Aorta" OR "aorta" OR "aortas" OR "aortic" OR "great blood vessel")) OR "Arterial Switch Operation" OR "atrial switch" OR "arterial switch" OR "atrial switch*" OR "arterial switch*" OR "double switch operation" OR "double switch procedure" OR "double switch technique" OR "double switch operations" OR "double switch procedures" OR "double switch" OR "jatene operation" OR "jatene procedure" OR "jatene technique" OR "mustard operation" OR "mustard procedure" OR "mustard repair" OR "rastelli operation" OR "rastelli procedure" OR "rastelli repair" OR "rastelli technique" OR "senning operation" OR "senning procedure" OR "jatene" OR "mustard child*" OR "mustard patient*" OR "rastelli" OR "senning") AND ("Fibrosis" OR "heart muscle fibrosis" OR "fibrosis" OR "fibroses" OR "fibrotic" OR "fibros*" OR "fibrot*" OR "fibrous" OR "Fibroblast" OR "Heart Fibroblast" OR "fibroblast" OR "fibroblasts" OR "Myofibroblast" OR "myofibroblast" OR "myofibroblasts" OR "fibroblast*" OR "fibrocellular" OR "fibrocell*" OR "fibrocollagen*" OR "fibrocollagenous" OR "myocardial remodelling" OR "myocardial remodeling" OR "myocardium remodelling" OR "myocardium remodeling" OR "myocardial remodel*" OR "myocardium remodel*" OR "Heart Ventricl Remodeling" OR "Ventricular Remodelling" OR "Ventricular Remodeling" OR "Ventricle Remodelling" OR "Ventricle Remodeling" OR "Ventricular Remodel*" OR "Ventricle Remodel*" OR "cardiac remodelling" OR "cardiac remodeling" OR "cardiac remodel*")) **OR** (("Arterial Switch Operation" OR "atrial switch" OR "arterial switch" OR "atrial switch*" OR "arterial switch*" OR "double switch operation" OR "double switch procedure" OR "double switch technique" OR "double switch operations" OR "double switch procedures" OR "double switch" OR "jatene operation" OR "jatene procedure" OR "jatene technique" OR "mustard operation" OR "mustard procedure" OR "mustard repair" OR "rastelli operation" OR "rastelli procedure" OR "rastelli repair" OR "rastelli technique" OR "senning operation" OR "senning procedure" OR "jatene" OR "mustard child*" OR "mustard patient*" OR "rastelli" OR "senning") AND ("Histology" OR "histology" OR "histolog*" OR "histocyto*"))):ti,ab,kw

Emcare http://ovidsp.ovid.com/ovidweb.cgi?T=JS&NEWS=n&CSC=Y&PAGE=main&D=emcr

(((exp *"Great Vessels Transposition"/ OR "transposition of the great arteries".ti,ab OR "transposition of the great artery".ti,ab OR "transposition of great arteries".ti,ab OR "transposition of great artery".ti,ab OR "transposition of the great vessels".ti,ab OR "transposition of great vessels".ti,ab OR "transposition of great vessel".ti,ab OR "great vessels transposition".ti,ab OR "great arteries transposition".ti,ab OR "congenitally corrected transposition".ti,ab OR "congenitally corrected transpositions".ti,ab OR "double outlet right ventricle".ti,ab OR "double outlet right ventricles".ti,ab OR "taussig bing anomaly".ti,ab OR (("transposition".ti,ab OR "transpositions".ti,ab OR "transpos*".ti,ab OR ("TGA".ti,ab NOT "transient global amnesia".ti,ab)) AND ("great vessel".ti,ab OR "great vessels".ti,ab OR "great arteries".ti,ab OR "great artery".ti,ab OR "large vessel".ti,ab OR "large vessels".ti,ab OR "large arteries".ti,ab OR "large artery".ti,ab OR exp *"Aorta"/ OR "aorta".ti,ab OR "aortas".ti,ab OR "aortic".ti,ab OR exp *"great blood vessel"/)) OR exp *"Arterial Switch Operation"/ OR "atrial switch".ti,ab OR "arterial switch".ti,ab OR "atrial switch*".ti,ab OR "arterial switch*".ti,ab OR "double switch operation".ti,ab OR "double switch procedure".ti,ab OR "double switch technique".ti,ab OR "double switch operations".ti,ab OR "double switch procedures".ti,ab OR "double switch".ti,ab OR "jatene operation".ti,ab OR "jatene procedure".ti,ab OR "jatene technique".ti,ab OR "mustard operation".ti,ab OR "mustard procedure".ti,ab OR "mustard repair".ti,ab OR "rastelli operation".ti,ab OR "rastelli procedure".ti,ab OR

"rastelli repair".ti,ab OR "rastelli technique".ti,ab OR "senning operation".ti,ab OR "senning procedure".ti,ab OR "jatene".ti,ab OR "mustard child*".ti,ab OR "mustard patient*".ti,ab OR "rastelli".ti,ab OR "senning".ti,ab) AND (*"Fibrosis"/ OR *"heart muscle fibrosis"/ OR "fibrosis".ti,ab OR "fibroses".ti,ab OR "fibrotic".ti,ab OR "fibros*".ti,ab OR "fibrot*".ti,ab OR "fibrous".ti,ab OR *"Fibroblast"/ OR *"Heart Fibroblast"/ OR "fibroblast".ti,ab OR "fibroblasts".ti,ab OR *"Myofibroblast"/ OR "myofibroblast".ti,ab OR "myofibroblasts".ti,ab OR "fibroblast*".ti,ab OR "fibrocellular".ti,ab OR "fibrocell*".ti,ab OR "fibrocollagen*".ti,ab OR "fibrocollagenous".ti,ab OR "myocardial remodelling".ti,ab OR "myocardial remodeling".ti,ab OR "myocardium remodelling".ti,ab OR "myocardium remodeling".ti,ab OR "myocardial remodel*".ti,ab OR "myocardium remodel*".ti,ab OR *"Heart Ventricl Remodeling"/ OR "Ventricular Remodelling".ti,ab OR "Ventricular Remodeling".ti,ab OR "Ventricle Remodelling".ti,ab OR "Ventricle Remodeling".ti,ab OR "Ventricular Remodel*".ti,ab OR "Ventricle Remodel*".ti,ab OR "cardiac remodelling".ti,ab OR "cardiac remodeling".ti,ab OR "cardiac remodel*".ti,ab)) **OR** ((exp *"Arterial Switch Operation"/ OR "atrial switch".ti,ab OR "arterial switch".ti,ab OR "atrial switch*".ti,ab OR "arterial switch*".ti,ab OR "double switch operation".ti,ab OR "double switch procedure".ti,ab OR "double switch technique".ti,ab OR "double switch operations".ti,ab OR "double switch procedures".ti,ab OR "double switch".ti,ab OR "jatene operation".ti,ab OR "jatene procedure".ti,ab OR "jatene technique".ti,ab OR "mustard operation".ti,ab OR "mustard procedure".ti,ab OR "mustard repair".ti,ab OR "rastelli operation".ti,ab OR "rastelli procedure".ti,ab OR "rastelli repair".ti,ab OR "rastelli technique".ti,ab OR "senning operation".ti,ab OR "senning procedure".ti,ab OR "jatene".ti,ab OR "mustard child*".ti,ab OR "mustard patient*".ti,ab OR "rastelli".ti,ab OR "senning".ti,ab) AND (exp *"Histology"/ OR "histology".ti,ab OR "histolog*".ti,ab OR "histocyto*".ti,ab)))

Academic Search Premier
http://search.ebscohost.com/login.aspx?authtype=ip,uid&profile=lumc&defaultdb=aph

(((TI("Great Vessels Transposition" OR "transposition of the great arteries" OR "transposition of the great artery" OR "transposition of great arteries" OR "transposition of great artery" OR "transposition of the great vessels" OR "transposition of great vessels" OR "transposition of great vessel" OR "great vessels transposition" OR "great arteries transposition" OR "congenitally corrected transposition" OR "congenitally corrected transpositions" OR "double outlet right ventricle" OR "double outlet right ventricles" OR "taussig bing anomaly" OR (("transposition" OR "transpositions" OR "transpos*" OR ("TGA" NOT "transient global amnesia")) AND ("great vessel" OR "great vessels" OR "great arteries" OR "great artery" OR "large vessel" OR "large vessels" OR "large arteries" OR "large artery" OR "Aorta" OR "aorta" OR "aortas" OR "aortic" OR "great blood vessel")) OR "Arterial Switch Operation" OR "atrial switch" OR "arterial switch" OR "atrial switch*" OR "arterial switch*" OR "double switch operation" OR "double switch procedure" OR "double switch technique" OR "double switch operations" OR "double switch procedures" OR "double switch" OR "jatene operation" OR "jatene procedure" OR "jatene technique" OR "mustard operation" OR "mustard procedure" OR "mustard repair" OR "rastelli operation" OR "rastelli procedure" OR "rastelli repair" OR "rastelli technique" OR "senning operation" OR "senning procedure" OR "jatene" OR "mustard child*" OR "mustard patient*" OR "rastelli" OR "senning") OR SU("Great Vessels Transposition" OR "transposition of the great arteries" OR "transposition of the great artery" OR "transposition of great arteries" OR "transposition of great artery" OR "transposition of the great vessels" OR "transposition of great vessels" OR "transposition of great vessel" OR "great vessels transposition" OR "great arteries transposition" OR "congenitally corrected transposition" OR "congenitally corrected transpositions" OR "double outlet right ventricle" OR "double outlet right ventricles" OR "taussig bing anomaly" OR (("transposition" OR "transpositions" OR "transpos*" OR ("TGA" NOT "transient global amnesia")) AND ("great vessel" OR "great vessels" OR "great arteries" OR "great artery" OR "large vessel" OR "large vessels" OR "large arteries" OR "large

artery" OR "Aorta" OR "aorta" OR "aortas" OR "aortic" OR "great blood vessel")) OR "Arterial Switch Operation" OR "atrial switch" OR "arterial switch" OR "atrial switch*" OR "arterial switch*" OR "double switch operation" OR "double switch procedure" OR "double switch technique" OR "double switch operations" OR "double switch procedures" OR "double switch" OR "jatene operation" OR "jatene procedure" OR "jatene technique" OR "mustard operation" OR "mustard procedure" OR "mustard repair" OR "rastelli operation" OR "rastelli procedure" OR "rastelli repair" OR "rastelli technique" OR "senning operation" OR "senning procedure" OR "jatene" OR "mustard child*" OR "mustard patient*" OR "rastelli" OR "senning") OR KW("Great Vessels Transposition" OR "transposition of the great arteries" OR "transposition of the great artery" OR "transposition of great arteries" OR "transposition of great artery" OR "transposition of the great vessels" OR "transposition of great vessels" OR "transposition of great vessel" OR "great vessels transposition" OR "great arteries transposition" OR "congenitally corrected transposition" OR "congenitally corrected transpositions" OR "double outlet right ventricle" OR "double outlet right ventricles" OR "taussig bing anomaly" OR (("transposition" OR "transpositions" OR "transpos*" OR ("TGA" NOT "transient global amnesia")) AND ("great vessel" OR "great vessels" OR "great arteries" OR "great artery" OR "large vessel" OR "large vessels" OR "large arteries" OR "large artery" OR "Aorta" OR "aorta" OR "aortas" OR "aortic" OR "great blood vessel")) OR "Arterial Switch Operation" OR "atrial switch" OR "arterial switch" OR "atrial switch*" OR "arterial switch*" OR "double switch operation" OR "double switch procedure" OR "double switch technique" OR "double switch operations" OR "double switch procedures" OR "double switch" OR "jatene operation" OR "jatene procedure" OR "jatene technique" OR "mustard operation" OR "mustard procedure" OR "mustard repair" OR "rastelli operation" OR "rastelli procedure" OR "rastelli repair" OR "rastelli technique" OR "senning operation" OR "senning procedure" OR "jatene" OR "mustard child*" OR "mustard patient*" OR "rastelli" OR "senning") OR AB("Great Vessels Transposition" OR "transposition of the great arteries" OR "transposition of the great artery" OR "transposition of great arteries" OR "transposition of great artery" OR "transposition of the great vessels" OR "transposition of great vessels" OR "transposition of great vessel" OR "great vessels transposition" OR "great arteries transposition" OR "congenitally corrected transposition" OR "congenitally corrected transpositions" OR "double outlet right ventricle" OR "double outlet right ventricles" OR "taussig bing anomaly" OR (("transposition" OR "transpositions" OR "transpos*" OR ("TGA" NOT "transient global amnesia")) AND ("great vessel" OR "great vessels" OR "great arteries" OR "great artery" OR "large vessel" OR "large vessels" OR "large arteries" OR "large artery" OR "Aorta" OR "aorta" OR "aortas" OR "aortic" OR "great blood vessel")) OR "Arterial Switch Operation" OR "atrial switch" OR "arterial switch" OR "atrial switch*" OR "arterial switch*" OR "double switch operation" OR "double switch procedure" OR "double switch technique" OR "double switch operations" OR "double switch procedures" OR "double switch" OR "jatene operation" OR "jatene procedure" OR "jatene technique" OR "mustard operation" OR "mustard procedure" OR "mustard repair" OR "rastelli operation" OR "rastelli procedure" OR "rastelli repair" OR "rastelli technique" OR "senning operation" OR "senning procedure" OR "jatene" OR "mustard child*" OR "mustard patient*" OR "rastelli" OR "senning")) AND (TI("Fibrosis" OR "heart muscle fibrosis" OR "fibrosis" OR "fibroses" OR "fibrotic" OR "fibros*" OR "fibrot*" OR "fibrous" OR "Fibroblast" OR "Heart Fibroblast" OR "fibroblast" OR "fibroblasts" OR "Myofibroblast" OR "myofibroblast" OR "myofibroblasts" OR "fibroblast*" OR "fibrocellular" OR "fibrocell*" OR "fibrocollagen*" OR "fibrocollagenous" OR "myocardial remodelling" OR "myocardial remodeling" OR "myocardium remodelling" OR "myocardium remodeling" OR "myocardial remodel*" OR "myocardium remodel*" OR "Heart Ventricl Remodeling" OR "Ventricular Remodelling" OR "Ventricular Remodeling" OR "Ventricle Remodelling" OR "Ventricle Remodeling" OR "Ventricular Remodel*" OR "Ventricle Remodel*" OR "cardiac remodelling" OR "cardiac remodeling" OR "cardiac remodel*") OR SU("Fibrosis" OR "heart muscle fibrosis" OR "fibrosis" OR "fibroses" OR "fibrotic" OR "fibros*" OR "fibrot*" OR "fibrous" OR "Fibroblast" OR "Heart Fibroblast" OR "fibroblast" OR "fibroblasts" OR "Myofibroblast" OR "myofibroblast" OR "myofibroblasts" OR "fibroblast*" OR "fibrocellular" OR "fibrocell*" OR "fibrocollagen*" OR "fibrocollagenous" OR "myocardial remodelling" OR "myocardial remodeling" OR "myocardium remodelling" OR "myocardium remodeling" OR

"myocardial remodel*" OR "myocardium remodel*" OR "Heart Ventricl Remodeling" OR "Ventricular Remodelling" OR "Ventricular Remodeling" OR "Ventricle Remodelling" OR "Ventricle Remodeling" OR "Ventricular Remodel*" OR "Ventricle Remodel*" OR "cardiac remodelling" OR "cardiac remodeling" OR "cardiac remodel*") OR KW("Fibrosis" OR "heart muscle fibrosis" OR "fibrosis" OR "fibroses" OR "fibrotic" OR "fibros*" OR "fibrot*" OR "fibrous" OR "Fibroblast" OR "Heart Fibroblast" OR "fibroblast" OR "fibroblasts" OR "Myofibroblast" OR "myofibroblast" OR "myofibroblasts" OR "fibroblast*" OR "fibrocellular" OR "fibrocell*" OR "fibrocollagen*" OR "fibrocollagenous" OR "myocardial remodelling" OR "myocardial remodeling" OR "myocardium remodelling" OR "myocardium remodeling" OR "myocardial remodel*" OR "myocardium remodel*" OR "Heart Ventricl Remodeling" OR "Ventricular Remodelling" OR "Ventricular Remodeling" OR "Ventricle Remodelling" OR "Ventricle Remodeling" OR "Ventricular Remodel*" OR "Ventricle Remodel*" OR "cardiac remodelling" OR "cardiac remodeling" OR "cardiac remodel*") OR AB("Fibrosis" OR "heart muscle fibrosis" OR "fibrosis" OR "fibroses" OR "fibrotic" OR "fibros*" OR "fibrot*" OR "fibrous" OR "Fibroblast" OR "Heart Fibroblast" OR "fibroblast" OR "fibroblasts" OR "Myofibroblast" OR "myofibroblast" OR "myofibroblasts" OR "fibroblast*" OR "fibrocellular" OR "fibrocell*" OR "fibrocollagen*" OR "fibrocollagenous" OR "myocardial remodelling" OR "myocardial remodeling" OR "myocardium remodelling" OR "myocardium remodeling" OR "myocardial remodel*" OR "myocardium remodel*" OR "Heart Ventricl Remodeling" OR "Ventricular Remodelling" OR "Ventricular Remodeling" OR "Ventricle Remodelling" OR "Ventricle Remodeling" OR "Ventricular Remodel*" OR "Ventricle Remodel*" OR "cardiac remodelling" OR "cardiac remodeling" OR "cardiac remodel*"))) **OR** ((TI("Arterial Switch Operation" OR "atrial switch" OR "arterial switch" OR "atrial switch*" OR "arterial switch*" OR "double switch operation" OR "double switch procedure" OR "double switch technique" OR "double switch operations" OR "double switch procedures" OR "double switch" OR "jatene operation" OR "jatene procedure" OR "jatene technique" OR "mustard operation" OR "mustard procedure" OR "mustard repair" OR "rastelli operation" OR "rastelli procedure" OR "rastelli repair" OR "rastelli technique" OR "senning operation" OR "senning procedure" OR "jatene" OR "mustard child*" OR "mustard patient*" OR "rastelli" OR "senning") OR SU("Arterial Switch Operation" OR "atrial switch" OR "arterial switch" OR "atrial switch*" OR "arterial switch*" OR "double switch operation" OR "double switch procedure" OR "double switch technique" OR "double switch operations" OR "double switch procedures" OR "double switch" OR "jatene operation" OR "jatene procedure" OR "jatene technique" OR "mustard operation" OR "mustard procedure" OR "mustard repair" OR "rastelli operation" OR "rastelli procedure" OR "rastelli repair" OR "rastelli technique" OR "senning operation" OR "senning procedure" OR "jatene" OR "mustard child*" OR "mustard patient*" OR "rastelli" OR "senning") OR KW("Arterial Switch Operation" OR "atrial switch" OR "arterial switch" OR "atrial switch*" OR "arterial switch*" OR "double switch operation" OR "double switch procedure" OR "double switch technique" OR "double switch operations" OR "double switch procedures" OR "double switch" OR "jatene operation" OR "jatene procedure" OR "jatene technique" OR "mustard operation" OR "mustard procedure" OR "mustard repair" OR "rastelli operation" OR "rastelli procedure" OR "rastelli repair" OR "rastelli technique" OR "senning operation" OR "senning procedure" OR "jatene" OR "mustard child*" OR "mustard patient*" OR "rastelli" OR "senning") OR AB("Arterial Switch Operation" OR "atrial switch" OR "arterial switch" OR "atrial switch*" OR "arterial switch*" OR "double switch operation" OR "double switch procedure" OR "double switch technique" OR "double switch operations" OR "double switch procedures" OR "double switch" OR "jatene operation" OR "jatene procedure" OR "jatene technique" OR "mustard operation" OR "mustard procedure" OR "mustard repair" OR "rastelli operation" OR "rastelli procedure" OR "rastelli repair" OR "rastelli technique" OR "senning operation" OR "senning procedure" OR "jatene" OR "mustard child*" OR "mustard patient*" OR "rastelli" OR "senning")) AND (TI("Histology" OR "histology" OR "histolog*" OR "histocyto*") OR SU("Histology" OR "histology" OR "histolog*" OR "histocyto*") OR KW("Histology" OR "histology" OR "histolog*" OR "histocyto*")OR AB("Histology" OR "histology" OR "histolog*" OR "histocyto*"))))

Search startegy Innervation:

PubMed
http://www.ncbi.nlm.nih.gov/pubmed?otool=leiden

Arterial Switch Operation & innervation/blood flow & imaging: 65 referenties d.d. 10-2-2021, incl. de 5 bekende (zonder de imaging component: 92 referenties).

(("Arterial Switch Operation"[Mesh] OR "Arterial Switch Operation"[tw] OR "Arterial Switch Operations"[tw] OR "arterial switch procedure"[tw] OR "arterial switch procedures"[tw] OR "arterial switch repair"[tw] OR "arterial switch surgery"[tw] OR "arterial switch"[tw] OR "Arterial Switch Technique"[tw] OR "Double Switch Operation"[tw] OR "Double Switch Operations"[tw] OR "Double Switch Procedure"[tw] OR "Double Switch Procedures"[tw] OR "Double Switch Technique"[tw] OR "Double Switch"[tw] OR "Jatene Operation"[tw] OR "Jatene Procedure"[tw] OR "Jatene Technique"[tw]) AND ("innervation"[subheading] OR "Heart/innervation"[Mesh] OR "innervation"[tw] OR "innervat*"[tw] OR "reinnervation"[tw] OR "reinnervat*"[tw] OR "Denervation"[Mesh] OR "denervation"[tw] OR "denerv*"[tw] OR "cardiac autonomic nerve"[tw] OR "autonomic cardiac nerve"[tw] OR "Cardiac sympathetic nerve"[tw] OR "sympathetic cardiac nerve"[tw] OR "sympathetic nerve"[tw] OR "autonomic nerve"[tw] OR "cardiac autonomic nerves"[tw] OR "autonomic cardiac nerves"[tw] OR "Cardiac sympathetic nerves"[tw] OR "sympathetic cardiac nerves"[tw] OR "sympathetic nerves"[tw] OR "autonomic nerves"[tw] OR "Sympathetic Nervous System"[mesh] OR "Autonomic Nervous System"[Mesh] OR "Nervous System"[Mesh] OR "Nervous System"[tw] OR "myocardial blood flow"[tw] OR "Coronary Circulation"[mesh] OR "coronary flow reserve"[tw]
OR " Autonomic dysfunction"[tw]
OR " Heart rate variability"[tw]
OR " Vasomotor (re)activity"[tw]
OR " Respiratory Sinus arrhythmia "[tw]
OR " Baroreflex sensitivity"[tw]
OR " The pre-ejection period "[tw]
OR " MIBG"[tw]
OR " Heart rate recovery"[tw]
OR " Heart rate reserve"[tw]
OR " QT variability"[tw])
AND ("Diagnostic Imaging"[mesh] OR "Diagnostic imaging"[subheading] OR "imaging"[tw] OR "PET"[tw] OR "positron emission"[tw] OR "perfusion"[tw] OR "adenosine"[tw] OR "echography"[tw] OR "ultrasound"[tw] OR "ultrasonography"[tw] OR "CT"[tw] OR "MRI"[tw] OR "MR imaging"[tw] OR "magnetic resonance"[tw] OR "perfusion*"[tw] OR "adenosine*"[tw] OR "echogra*"[tw] OR "ultrason*"[tw] OR "sonography"[tw] OR "sonogra*"[tw] OR "echocardiography"[tw] OR "echocardiogra*"[tw] OR "MR imag*"[tw]))

Autonomic dysfunction
Heart rate variability
Vasomotor (re)activity
Respiratory Sinus ***arrhythmia*** (parasympatische activiteit)
Baroreflex sensitivity
The pre-ejection period
MIBG
Heart rate recovery
Heart rate reserve
QT variability

Embase
http://ovidsp.ovid.com/ovidweb.cgi?T=JS&PAGE=main&MODE=ovid&D=oemezd

(*"Arterial Switch Operation"/ OR "Arterial Switch Operation"." OR "Arterial Switch Operations".ti,ab OR "arterial switch procedure".ti,ab OR "arterial switch procedures".ti,ab OR "arterial switch repair".ti,ab OR "arterial switch surgery".ti,ab OR "arterial switch".ti,ab OR "Arterial Switch Technique".ti,ab OR "Double Switch Operation".ti,ab OR "Double Switch Operations".ti,ab OR "Double Switch Procedure".ti,ab OR "Double Switch Procedures".ti,ab OR "Double Switch Technique".ti,ab OR "Double Switch".ti,ab OR "Jatene Operation".ti,ab OR "Jatene Procedure".ti,ab OR "Jatene Technique".ti,ab)

Web of Science
http://isiknowledge.com/wos

TS=("Arterial Switch Operation" OR "Arterial Switch Operation" OR "Arterial Switch Operations" OR "arterial switch procedure" OR "arterial switch procedures" OR "arterial switch repair" OR "arterial switch surgery" OR "arterial switch" OR "Arterial Switch Technique" OR "Double Switch Operation" OR "Double Switch Operations" OR "Double Switch Procedure" OR "Double Switch Procedures" OR "Double Switch Technique" OR "Double Switch" OR "Jatene Operation" OR "Jatene Procedure" OR "Jatene Technique") AND TS=("innervation" OR "innervat*" OR "reinnervation" OR "reinnervat*" OR "Denervation" OR "denervation" OR "denerv*" OR "cardiac autonomic nerve" OR "autonomic cardiac nerve" OR "Cardiac sympathetic nerve" OR "sympathetic cardiac nerve" OR "sympathetic nerve" OR "autonomic nerve" OR "cardiac autonomic nerves" OR "autonomic cardiac nerves" OR "Cardiac sympathetic nerves" OR "sympathetic cardiac nerves" OR "sympathetic nerves" OR "autonomic nerves" OR "Sympathetic Nervous System")

Cochrane
https://www.cochranelibrary.com/advanced-search/search-manager

("Arterial Switch Operation" OR "Arterial Switch Operation" OR "Arterial Switch Operations" OR "arterial switch procedure" OR "arterial switch procedures" OR "arterial switch repair" OR "arterial switch surgery" OR "arterial switch" OR "Arterial Switch Technique" OR "Double Switch Operation" OR "Double Switch Operations" OR "Double Switch Procedure" OR "Double Switch Procedures" OR "Double Switch Technique" OR "Double Switch" OR "Jatene Operation" OR "Jatene Procedure" OR "Jatene Technique"):ti,ab,kw

Emcare http://ovidsp.ovid.com/ovidweb.cgi?T=JS&NEWS=n&CSC=Y&PAGE=main&D=emcr

GoogleScholar

"Arterial Switch"|"Double Switch Operation"|"Double Switch"|"Jatene" "innervation"|"reinnervation"

"Denervation"|"denervation"|"denerv*"|"cardiac autonomic nerve"|"autonomic cardiac nerve"|"Cardiac sympathetic nerve"|"sympathetic cardiac nerve"|"sympathetic nerve"|"autonomic nerve"|"cardiac autonomic nerves"|"autonomic cardiac nerves"|"Cardiac sympathetic

nerves"|"sympathetic cardiac nerves"|"sympathetic nerves"|"autonomic nerves"|"Sympathetic Nervous System
